# Design and Functionality of Trypsin‐Triggered, Expandable Bovine Serum Albumin‐Polyethylene Glycol Diacrylate Hydrogel Actuators

**DOI:** 10.1002/smsc.202400214

**Published:** 2024-07-21

**Authors:** Yuchen Liu, Luai R. Khoury

**Affiliations:** ^1^ Department of Materials Science and Engineering Technion Israel Institute of Technology Haifa 32000 Israel

**Keywords:** albumin, controlled drug release, polyethylene glycol diacrylate, protein‐based materials, soft actuators

## Abstract

Expandable shape‐morphing hydrogels that ensure prolonged site residence, have tailored mechanical integrity and tunability, are biocompatible to minimize side effects and can release drugs over an extended time remain challenging to achieve. Herein, a new class of enzyme‐triggered bovine serum albumin and polyethylene glycol diacrylate hybrid hydrogels is presented, contributing to advancements in controlled drug‐model release and actuation. These hydrogels combine the intrinsic properties of proteins with the resilience of synthetic polymers, offering a versatile application platform. Central to our research is the trypsin‐induced simultaneous functionality of controlled drug model release and dynamic shape changes under physiological trypsin concentrations (0.01% w/v). These hydrogels display tailored mechanical and physical properties and microstructure, which are crucial for biomedical devices, soft robotics, and tissue engineering applications. Additionally, the hydrogels effectively control the release of fluorescein isothiocyanate, a model drug, indicating their potential for highly targeted drug delivery, particularly in the gastrointestinal tract. The study also highlights the significant effect of shape‐morphing on drug release rates under physiological trypsin concentrations. These findings suggest that enzyme‐responsive hybrid protein‐polymer hydrogel actuators with tailored mechanical and physical properties can enhance the precision of drug delivery in biomedical applications.

## Graphical abstract

This study presents the design and functionality of trypsin‐triggered, expandable bovine serum albumin‐polyethylene glycol diacrylate (BSA‐PEGDA) hydrogel actuators. Integrating BSA with PEGDA achieved enzyme‐specific shape morphing and controlled fluorescein isothiocyanate (FITC) release hydrogels. The hydrogels tunable mechanical characteristics and controlled microstructure, making them suitable for biomedical applications such as tissue engineering and targeted drug delivery.

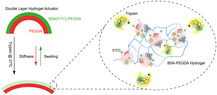

## 1 Introduction

In the intersection of materials science and biomedicine, stimuli‐responsive hydrogels have shown potential for precision in drug delivery systems and actuators. The unique properties of hydrogels, including superior biocompatibility and intricate porous structures, make them advantageous in drug delivery systems and tissue engineering.^[^ ^1^, ^2^, ^3^ ^]^ Their ability to respond to environmental triggers, such as changes in pH,^[^ ^4^, ^5^, ^6^, ^7^, ^8^ ^]^ temperature,^[^ ^4^, ^9^, ^10^, ^11^ ^]^ and specific enzymes,^[^ ^12^, ^13^ ^]^ is crucial for controlled drug release, ensuring dosage accuracy while minimizing risks linked to elevated plasma drug concentrations. This responsiveness transforms them from static materials into environmentally responsive materials, which supports developing actuators.

Hydrogels are fundamentally hydrophilic polymers with a three‐dimensional network structure.^[^ ^1^, ^14^ ^]^ Their adaptability, achieved by altering their constituent components, enables various applications, such as drug delivery^[^ ^1^, ^14^, ^15^ ^]^ and tissue engineering scaffolds.^[^ ^16^, ^17^ ^]^ These polymers can be either naturally derived, such as alginate^[^ ^18^, ^19^ ^]^ or chitosan,^[^ ^20^, ^21^ ^]^ known for their excellent biocompatibility, or synthetic, such as polyethylene glycol diacrylate (PEGDA),^[^ ^22^, ^23^ ^]^ poly (N‐isopropyl acrylamide) (PNIPAM),^[^ ^24^, ^25^ ^]^ and polyacrylic acid (PAA),^[^ ^26^, ^27^ ^]^ which are praised for their superior mechanical properties. However, naturally derived hydrogels often lack mechanical strength in demanding physiological environments, whereas synthetic hydrogels may have reduced bioactivity and biocompatibility.

Peptide‐ and nonglobular protein‐based hydrogels are increasingly used in biomedical research. Studies focus on manipulating peptide chains and structuring alpha‐helix and beta‐sheet formations to enhance peptide‐based hydrogels’ mechanical and microstructural characteristics.^[^ ^28^, ^29^, ^30^, ^31^ ^]^ In contrast, globular protein‐based hydrogels show distinct viscoelasticity, relaxation times, and functionality influenced by protein domain folding at the nanoscale.^[^ ^32^, ^33^, ^34^, ^35^, ^36^, ^37^, ^38^ ^]^ These hydrogels exhibit shape‐memory and morphing abilities due to protein (un)folding nanomechanics.^[^ ^39^, ^40^, ^41^ ^]^ Additionally, protein engineering is employed to create muscle‐like biomaterials and self‐healing hydrogels,^[^ ^42^, ^43^ ^]^ utilizing protein biochemical diversity and its interaction with polymers and cations to customize hydrogel mechanics. Still, they might not always rival the robustness of synthetic hydrogels.

A notable advancement is the development of hybrid protein‐polymer hydrogels,^[^ ^44^, ^45^, ^46^, ^47^, ^48^ ^]^ representing a synergy of biocompatibility and mechanical robustness. These hybrids utilize the intrinsic biocompatibility and biochemical‐structural diversity of proteins, along with their unique folding and unfolding nanomechanics, and merge them with the durability and stability of synthetic polymers for various bioapplications, such as self‐healing hydrogels,^[^ ^46^ ^]^ 3D printing tissue scaffolds,^[^ ^45^ ^]^ and adjustable protein‐based lens.^[^ ^49^ ^]^

With their intricate three‐dimensional frameworks, responsive hydrogels have gained prominence for their ability to adjust to external cues like pH, temperature, light, or strain.^[^ ^50^ ^]^ Their dynamic adaptability has paved the way for drug delivery and soft robotic advancements.^[^ ^47^, ^48^ ^]^ However, the quest for optimal activation stimuli of hydrogels persists. Traditional stimuli like light, heat, pH, and magnetism carry inherent limitations within the human body, such as potential tissue damage or limited tissue penetration.^[^ ^51^, ^52^ ^]^ Within this field, enzyme‐responsive hydrogels emerge as a promising solution.^[^ ^12^, ^13^, ^53^, ^54^, ^55^, ^56^, ^57^ ^]^ They are designed to structurally respond to specific enzymes, making them particularly suitable for therapeutic applications in tumors, inflammatory sites, or gastrointestinal (GI) tract conditions where certain enzymes are overexpressed.^[^ ^58^, ^59^, ^60^ ^]^ For example, enzyme‐responsive microgrippers use selective enzymatic degradation of biopolymer hinges to open and close within the liver.^[^ ^57^ ^]^ Other approaches employ collagenase–gelatin interactions to create folding bilayer hydrogel actuators.^[^ ^54^ ^]^ Additionally, cytocompatible shape memory polymers respond to enzymatic activity, using poly(e‐caprolactone) for shape fixing and Pellethane for shape memory.^[^ ^61^ ^]^ Recently, catalase‐catalyzed oxygen gas generation has been used to operate a pneumatically driven soft robot.^[^ ^56^ ^]^ Nevertheless, an expandable enzyme‐triggered shape‐morphing biocompatible hydrogel for prolonged site residence, with mechanical integrity and tunability, controlled microstructure, and the capability to be drug‐loaded and enzymatically released over a prolonged time, remains a challenging goal.

In this study, we explore the protein–enzyme digestive interactions between bovine serum albumin (BSA) and trypsin to introduce a tunable enzyme‐triggered shape‐morphing hybrid BSA‐PEGDA hydrogel actuator. This system is designed to control the release of fluorescein isothiocyanate (FITC) as a model compound, showcasing its potential for precise delivery applications. By modulating PEGDA and BSA concentrations, we achieved controlled hydrogel stiffness. Furthermore, trypsin treatment enabled us to fine‐tune the material's microstructure and mechanical behavior. The hydrogel samples were synthesized by combining BSA‐PEGDA or PEGDA solution with ammonium persulfate (APS) and tetramethylethylenediamine (TEMED). Our system's fundamental design involves an arc‐shaped bilayer hydrogel actuator made from two layers of BSA‐PEGDA (2–100 mM) and PEGDA (200 mM) hydrogels. Exposing the bilayer actuator to trypsin decreased stiffness and increased swelling, exerting enough force to create a shape‐morphing behavior. Another important result is that we harnessed the diverse biochemical structures of BSA to load covalently FITC, a model for a small molecule drug, on the BSA surface. By manipulating different trypsin concentrations and varying hydrogel cross‐linking network densities, we achieved intelligent and controlled release of FITC. Finally, combining both approaches, we created a soft robotic drug carrier and found that shape changes significantly influenced FITC release rates under physiological trypsin concentrations (0.01% w/v). This broadens the utility of BSA's role to enzyme‐triggered shape‐morphing actuators and simultaneously as an active carrier capable of manipulating drug release in response to enzymatic digestion. Considering the latest findings, this method is expected to be utilized in various biological applications, particularly in targeted drug delivery and bariatric procedures within a wide spectrum of GI applications.

## 2 Results and Discussion

### 2.1 Controlled Tuning of BSA‐PEGDA Hydrogel Physical Properties via BSA‐Trypsin Interactions

**Figure 1:**
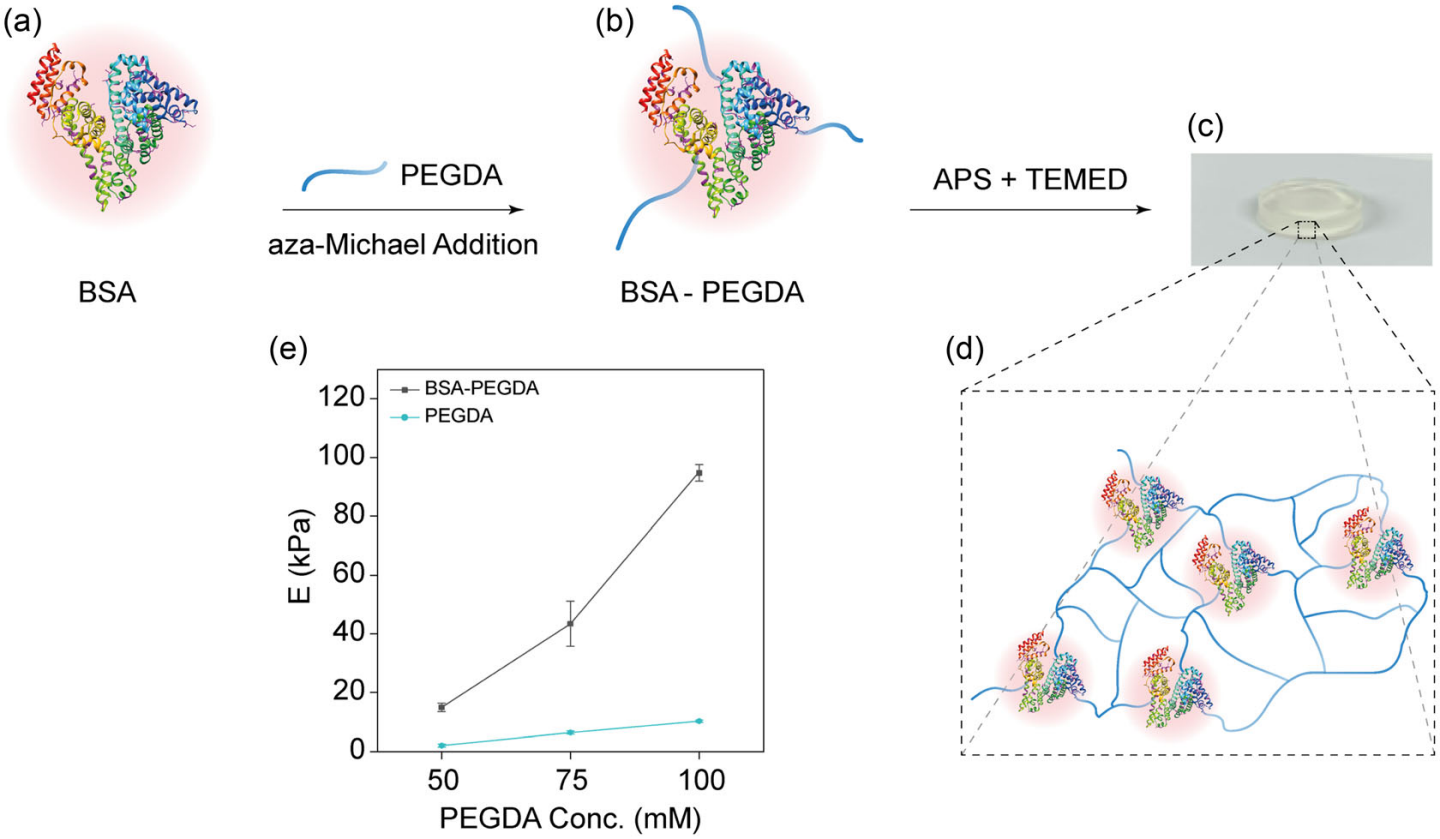
Synthesis and characterization of BSA‐PEGDA hydrogels. a) BSA and PEGDA are mixed under magnetic stirring overnight. This step leverages an aza‐Michael addition reaction for bioconjugation, where lysine residues’ free amino groups undergo a reaction with PEGDA's vinyl groups. b) The formed BSA‐PEGDA mixture is then introduced into a free radical‐generating system containing APS (1 M) and TEMED (10 mM) solutions at a specific volume ratio of (9:1:10), respectively. Within this system, TEMED and APS play a crucial role in initiating the polymerization reaction of PEGDA, leading to the hydrogel formation. c) A close‐up image of the BSA‐PEGDA hydrogel sample. d) A scheme depicting the cross‐linked network within the BSA‐PEGDA hydrogel shows the incorporation of BSA into the PEGDA network. e) BSA‐PEGDA and PEGDA hydrogels were formulated with varying concentrations of PEGDA (50, 75, and 100 mM). The samples underwent compression tests at a rate of 1 mm min^−1^. Young's modulus was determined based on the slope of the stress–strain curve within the 0–10% range. It was observed that Young's modulus escalated in both hydrogel types as the concentration of PEGDA was raised. This increase was particularly more pronounced in the BSA‐PEGDA hydrogel samples.

Incorporating protein molecules into polymer hydrogel networks has significantly improved the material's mechanical behavior, optical transparency, and functionality.^[^ ^49^, ^62^ ^]^ To explore this, we used BSA, functionalized with PEGDA via aza‐Michael addition, which forms covalent bonds between the amino groups of BSA and the acrylate functional groups of PEGDA (**Figure** **1**a,b).^[^ ^47^, ^49^ ^]^ BSA was selected due to its high stability, diverse biochemical structure and flexibility, and low price compared to other proteins.^[^ ^63^, ^64^, ^65^, ^66^ ^]^ Following this, the BSA‐PEGDA mixture was then mixed with APS (1 M) and TEMED (10 mM) at 9: 1: 10 volume ratios for 5 min at room temperature (RT) to form BSA‐PEGDA hydrogel through a free radical generation system, initiating the polymerization of the vinyl groups of different PEGDA molecules crosslinked through carbon‐carbon covalent bonds (Figure 1c,d). Subsequently, our investigation focused on understanding the influence of the BSA to PEGDA concentration ratio on the hydrogel network's mechanical characteristics. For this purpose, we conducted a compressive test, applying a 1 mm min^−1^ rate, to evaluate these effects comprehensively. Our results revealed that increasing the PEGDA concentration (50, 75, 100 mM) while maintaining the BSA concentration constant (2 mM) significantly increased the hydrogel stiffness. Conversely, PEGDA hydrogel samples prepared with similar PEGDA concentrations but without BSA showed only a slightly increased stiffness (Figure 1e, Supporting Figure 1, Supporting Information). These observations are likely due to the increase in lysine residue functionalization associated with higher PEGDA concentration and the BSA folding transitions.^[^ ^49^ ^]^

**Figure 2:**
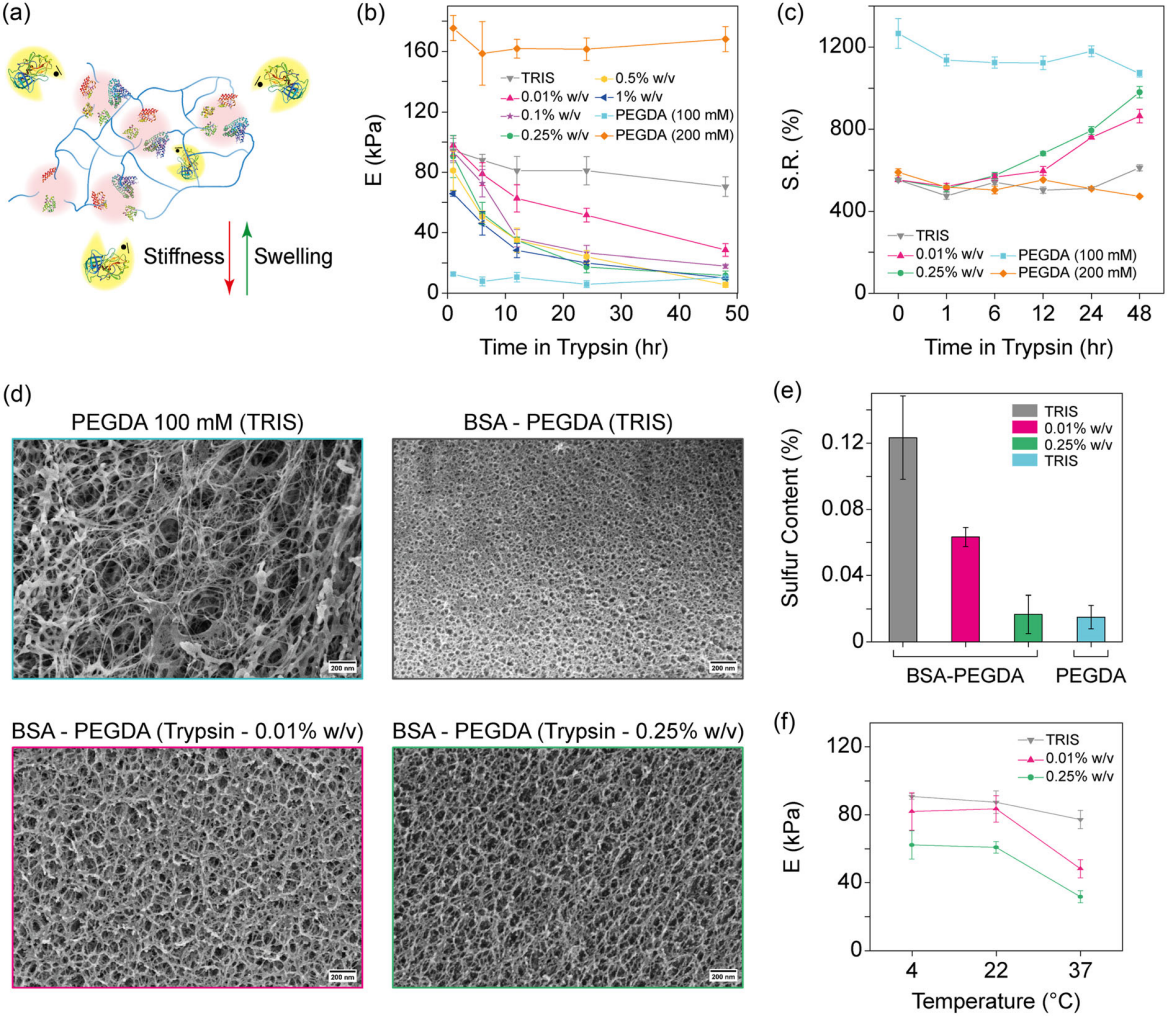
Characterization of BSA‐PEGDA hydrogels: mechanical integrity, enzymatic degradation, and swelling dynamics. a) A schematic illustration demonstrating the BSA‐PEGDA microstructure post‐trypsin exposure, depicting BSA degradation and subsequent release from the hydrogel matrix. b) BSA‐PEGDA (2–100 mM) hydrogel samples were immersed in trypsin solution at 37 °C at various concentrations (0.01, 0.1, 0.25, 0.5, and 1%, w/v) for different periods (1, 6, 12, 24, and 48 h). Trypsin‐induced degradation of BSA into the hydrogel samples led to a consistent decrease in Young's modulus. The reduction was dependent on both enzyme concentration and exposure time. Initially, stiffness declined proportionally to the increase in trypsin concentration, but over time, the response plateaued for concentrations above 0.1%, stabilizing after 48 h to levels comparable to those of PEGDA (100 mM). The lowest trypsin concentration (0.01% w/v) resulted in a minor change in stiffness, reflecting the minimal enzyme activity. In TRIS, a slight decrease was observed over time. c) SR measurements of BSA‐PEGDA and PEGDA hydrogels immersed in trypsin (0.01% or 0.25% w/v) or TRIS for different durations at 37 °C. PEGDA and BSA‐PEGDA hydrogels showed a constant SR behavior over time in TRIS. BSA‐PEGDA hydrogel samples in trypsin showed increased swelling behavior over time, approaching that of PEGDA hydrogel (100 mM) after 48 h. d) Cryo‐SEM images of PEGDA and BSA‐PEGDA hydrogel samples immersed in TRIS and trypsin (0.01% or 0.25% w/v) for 48 h. PEGDA appeared disordered with large pore structures, and the framework exhibited fibrous characteristics. The cross‐linking structure became more ordered and compact after adding BSA protein to this hydrogel system, with pore structures reducing to ≈30 nm. Following trypsin treatment, the pore structure expanded to a maximum of around 200 nm, and the framework also adopted a fibrous appearance. However, different concentrations of trypsin did not significantly affect pore size, but the fiber thickness became finer at higher trypsin concentrations, scale bar is 200 nm. e) Variation in the content of sulfur element under different conditions. For 0.01%, the sulfur content had decreased to half of the initial sulfur content in the BSA‐PEGDA hydrogel. Meanwhile, for 0.25%, the sulfur content had dropped to the sulfur level in the PEGDA hydrogel, nearly approaching zero. f) BSA‐PEGDA was treated with trypsin (0.01% and 0.25% w/v) for 24 h at different temperatures (4, 22, and 37 °C). When BSA‐PEGDA hydrogels were immersed in trypsin, Young's modulus reduced with increasing temperature, and there was a slight decrease in TRIS. Still, higher trypsin concentrations caused a more significant decrease, notably. At 4 °C, the high trypsin concentration compensated for the reduced enzyme activity caused by the low temperature.

Given the recent findings emphasizing the BSA's role in enhancing the mechanical response of hybrid BSA‐PEGDA hydrogels, we proposed that a controlled elimination of BSA from the hydrogel matrix will enable fine‐tuning of the hydrogel stiffness and swelling ratio (SR) (**Figure** **2**a). Thus, we utilized the protein–enzyme digestive interaction and immersed the BSA‐PEGDA hydrogel samples in various concentrations of trypsin solutions (pH ≈ 7.7) ranging from 0 to 1% w/v for different intervals of 1, 6, 12, 24, and 48 h at 37 °C. We found that a BSA‐PEGDA hydrogel sample with a concentration of 2–100 mM and without trypsin treatment showed no significant change in Young's modulus after being subjected to a compressive mechanical test (1 mm min^−1^). However, the other BSA‐PEGDA hydrogels showed a noticeable decrease in stiffness over time. Furthermore, when observing the stiffness behavior of hydrogels subjected to different trypsin concentrations, it was evident that the hydrogel stiffness was highly sensitive to varying trypsin concentrations in the first hour. The higher the concentration, the faster the stiffness decreased. By the sixth hour, the changes in stiffness of the hydrogels were no longer sensitive to trypsin concentrations of 0.25%, 0.5%, and 1%, as their stiffness values were nearly identical. However, owing to its low enzyme catalytic efficiency, the 0.01% concentration group, resembling a normal physiological level,^[^ ^67^ ^]^ did not converge with the other groups over the entire duration (Figure 2b, Supporting Figure 2 and 3, Supporting Information). This reduction in stiffness can be attributed to the enzymatic degradation of BSA by trypsin, resulting in the elimination of the BSA folded structure role in enhancing the mechanical behavior of BSA‐PEGDA and the decrease in the crosslinking density within the hydrogel matrix, consequently leading to a softer hydrogel with stiffness approaching that of PEGDA after 48 h. Finally, after 48 h of exposure to trypsin (0.25% w/v), PEGDA (100 mM) hydrogels exhibited no significant changes in their mechanical properties, maintaining Young's modulus of around 10 kPa. Similarly, PEGDA (200 mM) hydrogel immersed in TRIS buffer (Tris 20 mM, NaCl 150 mM, pH ≈ 7.4) showed no significant alterations, maintaining Young's modulus of ≈170 kPa. This underscores the importance of the enzymatic hydrolysis of BSA in regulating hydrogel stiffness changes.

Additionally, the hydrogels’ swelling patterns aligned with changes in their crosslinking density and mechanical response (Figure 2c). Our observations of the PEGDA hydrogels, prepared at 100 or 200 mM concentrations, indicated a stable swelling response throughout the process. Conversely, BSA‐PEGDA hydrogels (2–100 mM) demonstrated a notable increase in their SRs following trypsin treatment, a change we attribute to the enzymatic breakdown and subsequent removal of BSA from the hydrogel network allowing for increasing water absorption. Specifically, hydrogels exposed to a 0.25% w/v trypsin concentration showed a higher SR than those treated with a lower concentration of 0.01% w/v. In addition, it was also found that for 48 h, the SR of the BSA‐PEGDA hydrogel in TRIS slightly increased.

To deepen our understanding of trypsin's influence on BSA‐PEGDA hydrogel samples, cryo‐scanning electron microscopy (cryo‐SEM) was utilized to observe changes in the hydrogel's microstructure and morphology pre‐ and post‐trypsin treatment. Cryo‐SEM images revealed that the PEGDA hydrogels (100 mM) possessed a disordered internal mesh with large pores and a fibrous texture (Figure 2d). In contrast, the microstructure of BSA‐PEGDA (2–100 mM) in the TRIS solution was denser, with a reduced average pore size of about 30 nm (Figure 2d). Notably, post‐trypsin treatment at both 0.01% and 0.25% w/v concentrations, the BSA‐PEGDA samples exhibited an increase in pore size to around 200 nm, alongside a transition to a more fibrous mesh. While the pore sizes between the two trypsin concentrations did not differ significantly, a distinctly thinner fibrous mesh characterized the 0.25% trypsin hydrogels (Figure 2d), which aligns with the mechanical properties and swelling behavior presented in Figure 2b,c.

Furthermore, we leveraged the unique, intricate biochemical composition of BSA, which includes 17 disulfide bonds, to track the digestion and subsequent removal of the protein from the hydrogel network. To this end, we utilized energy‐dispersive X‐ray spectroscopy (EDS) to analyze the presence of sulfur—a distinctive marker of the BSA structure—within the hydrogel. After 48 h (Figure 2e), the sulfur content in the BSA‐PEGDA hydrogel treated with 0.01% trypsin had decreased to roughly 50% of the initial level observed in the BSA‐PEGDA hydrogel control group. This suggests that not all fragments were released from the hydrogel while BSA was partially digested. Conversely, the sulfur levels in the BSA‐PEGDA samples immersed in 0.25% trypsin were nearly indistinguishable from those in PEGDA hydrogels, implying an almost complete degradation and release of BSA peptide fragments containing sulfur element from the hydrogel structure. The latter observations highlight the efficacy of trypsin in not only altering the physical properties of hydrogels but also in enabling the modulation of protein content within these materials. The near‐total elimination of BSA from hydrogels treated with higher trypsin concentrations underscores the potential of enzymatic treatments in tailoring the characteristics of protein‐incorporated hydrogels for specific applications. This capability could be particularly advantageous in developing hydrogels with customized mechanical properties for biomedical applications such as tissue engineering and drug delivery systems.

Due to the significant effect of temperature on trypsin activity,^[^ ^68^, ^69^ ^]^ we examined its influence on the mechanical properties of BSA‐PEGDA hydrogels. Our findings indicate a significant interplay between temperature, enzymatic activity, and enzyme concentration on hydrogel stiffness (Figure 2f). BSA‐PEGDA hydrogels immersed in 0.01% and 0.25% w/v trypsin solutions for 24 h exhibited decreased stiffness with increasing temperature, suggesting an enhanced trypsin enzymatic activity that leads to more substantial digestion of BSA in the hydrogel samples. Conversely, samples immersed solely in TRIS buffer displayed no significant stiffness changes across the temperature range, which aligns with the absence of enzymatic activity.

Furthermore, at a lower temperature of 4 °C, an increased trypsin concentration seemed to compensate for the reduced enzymatic activity commonly observed at low temperatures. Conversely, at 37 °C, the stiffness reduction occurred more rapidly for both trypsin concentrations, confirming that this temperature optimizes trypsin's enzymatic efficiency (Figure 2f).

### 2.2 Development and Characterization of Enzyme‐Responsive BSA‐PEGDA Hydrogel Actuators

**Figure 3:**
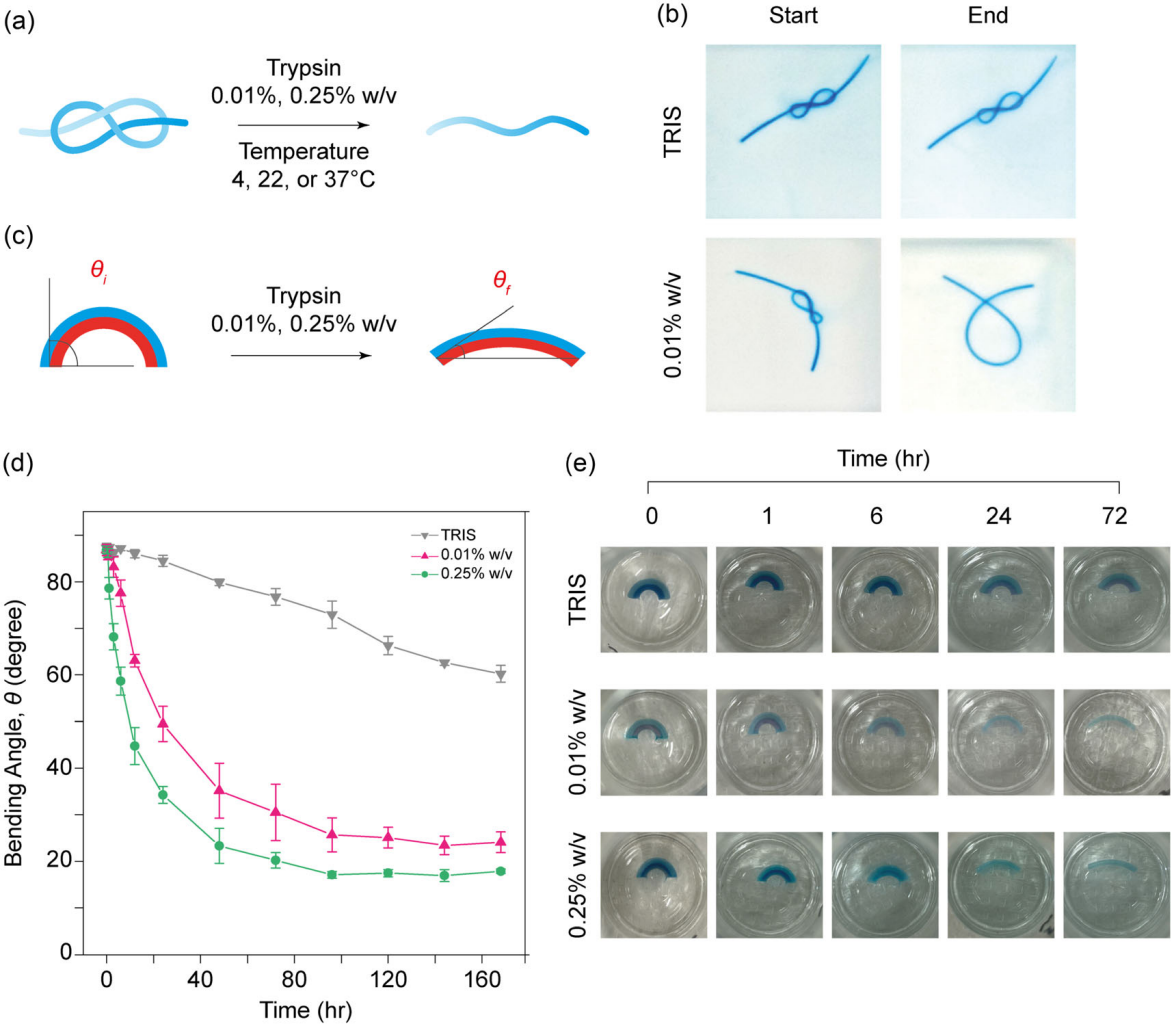
Enzyme‐induced shape‐morphing behavior of BSA‐PEGDA hydrogel actuators. a) A scheme illustrating wire‐like shape BSA‐PEGDA (2–100 mM) hydrogel sample fixed in the figure of eight shape “8” in TRIS. Subsequently, when the programmed “8” shape hydrogel sample is exposed to trypsin at 37 °C, the shape morphs back to its original shape. b) Close‐up images of the eight shapes “8” BSA‐PEGDA hydrogel samples in TRIS and trypsin. The images show that programmed BSA‐PEGDA hydrogel in TRIS does not change the shape. In contrast, the sample in trypsin changes and releases the “8” shape (Supporting Movie 1, Supporting Information). c) An illustration of bilayer arc‐shape PEGDA/BSA‐PEGDA hydrogel actuator before and after being exposed to trypsin. The bilayer actuator was formed as follows: an outer layer of PEGDA hydrogel (200 mM) (blue) was bent in a semicircular shape using a mold. Then, a BSA‐PEGDA layer (2–100 mM) (red) was added as an inner layer. After that, the bilayer actuator was released from the mold and immersed in TRIS. After exposure to trypsin and BSA degradation, the stiffness of the inner layer (BSA‐PEGDA) decreases, and the SR increases, leading to outer layer recovery to linear form, driving the shape‐morphing behavior of the bilayer actuator. The bending angle, *θ*, represents the deviation from the original arc shape. d) Bending angle measurements and e) close‐up images of bilayer arc‐shaped actuators immersed in TRIS and trypsin (0.01% or 0.25% w/v) over time at 37 °C. The bilayer actuator in TRIS had a slightly altered shape, while actuators in trypsin showed a decreased bending angle over time. The actuator in trypsin (0.25% w/v) shows a more notable reduction, emphasizing the effect of BSA in contributing to the hydrogel stiffness.

Leveraging the unique, enzyme‐responsive behavior of BSA‐PEGDA hydrogels, which we observed in their mechanical properties and SRs following BSA digestion (Figure 2), our study ventured into developing an enzyme‐induced hybrid protein‐polymer hydrogel actuator. Accordingly, we synthesized a wire‐like hydrogel sample and shaped the hydrogel samples into an eight‐like shape “8” (**Figure** **3**a). Then, the programmed hydrogel samples were immersed in TRIS and trypsin solutions (0.01% and 0.25% w/v) at various temperatures (4, 22, and 37 °C) (Figure 3a). Interestingly, we found no shape change for the programmed hydrogel in TRIS. However, the eight‐like hydrogel samples exposed to trypsin untied due to alterations in stiffness and swelling behavior (Figure 3b). In addition, the knot shape‐morphing response time varied with trypsin concentration, where a high trypsin concentration (0.25% w/v) resulted in higher enzymatic activity and, therefore, quicker shape‐morphing reactions compared with a low trypsin concentration (0.01% w/v). Furthermore, the shape‐morphing behavior also correlates with temperature, influencing enzyme activity (Figure 2f, 3b, and Supporting Movie 1, Supporting Information).

Expanding upon this idea, we fabricated a bilayer arc‐shaped actuator, which morphs its shape and straightens when exposed to trypsin (Figure 2c). First, we formed a straight rectangular PEGDA (200 mM) layer, which was then fixed into a semicircular shape in the outer section of a bilayer mold (Supporting Figure 4, Supporting Information). Then, we created the inner layer from BSA‐PEGDA (2–100 mM) hydrogel. Upon removal of the bilayer arc‐shaped actuator from the mold, the arc shape was preserved due to similar SRs and stiffness of the BSA‐PEGDA layer compared to PEGDA (Figure 2, 3c). The bilayer arc‐shaped hydrogel actuator was immersed in TRIS or trypsin solutions for 168 h at 37 °C.

The angle *θ*, reflecting the shift from the original arc configuration, steadily decreased in trypsin solutions of various concentrations (0.01 and 0.25% w/v). This decrease was more pronounced in the 0.25% w/v trypsin solution; the hydrogel actuator changed its shape from *θ*_i_ = 87 ± 1° to *θ*_f_ = 18 ± 1° within 72 h and remained stable thereafter. For 0.01%, the actuator's angle decreased from *θ*_i_ = 87 ± 1° to *θ*_f_ = 24 ± 1° (Figure 3d,e) within 96 h and remained stable thereafter. Such a pattern is likely a result of the concurrent increase in swelling behavior and decreases in BSA‐PEGDA stiffness due to the trypsin treatment. Over time, the swelling ability of the BSA‐PEGDA layer surpasses that of the PEGDA (200 mM) layer. The PEGDA (200 mM) hydrogel demonstrates significantly higher stiffness through trypsin treatment than the BSA‐PEGDA hydrogel (Figure 2b,c). This synergistic effect of decreased stiffness and increased swelling behavior allows the PEGDA hydrogel to exert sufficient force to revert to its original straight shape (Figure 3d). In TRIS, the hydrogel actuator changed its shape from *θ*_i_ = 87 ± 1° to *θ*_f_ = 60 ± 1° which can be attributed to the PEGDA degradation resulting in stiffness decrease over time.

### 2.3 Enzyme‐Triggered FITC Release from BSA‐PEGDA Hydrogel Actuators

**Figure 4:**
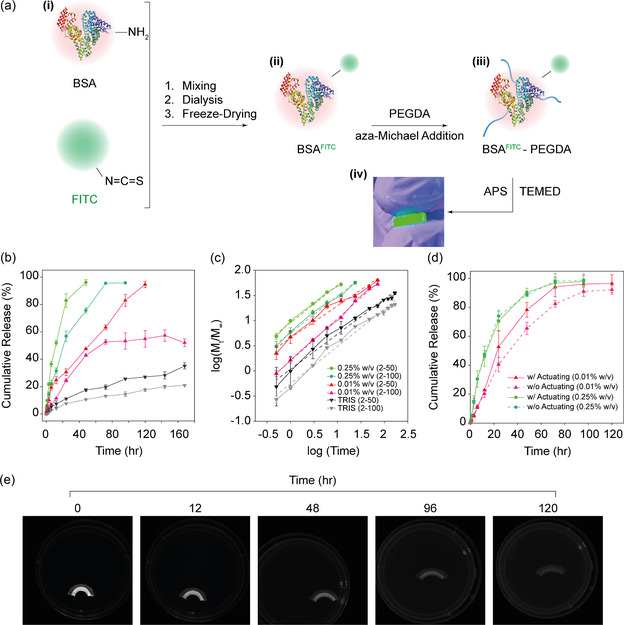
Synthesis and characterization of FITC‐conjugated BSA‐PEGDA hydrogels for enzymatically‐controlled drug release. a) BSA‐FITC conjugation for BSA(FITC)‐PEGDA hydrogel synthesis. i) Initial mixing of BSA with FITC leads to the covalent bonding of FITC's isothiocyanate groups to the amino groups on the lysine residues of BSA, resulting in a stable, green, fluorescent BSA(FITC) powder. ii) Subsequently, the BSA(FITC) is functionalized with PEGDA via an aza‐Michael addition reaction, wherein the remaining free amino groups of BSA lysine residues react with the vinyl groups of PEGDA. iii) This BSA(FITC)‐PEGDA mixture is then mixed with APS and TEMED in a volume ratio of 9:1:10, facilitating the formation of the BSA(FITC)‐PEGDA hydrogel. iv) A close‐up image of the resultant BSA(FITC)‐PEGDA hydrogel demonstrates the characteristic green fluorescence when exposed to ultraviolet light, confirming the successful bioconjugation of FITC to the hydrogel matrix. b) Cumulative release (%) curves of FITC from BSA(FITC)‐PEGDA (2–50, 2–100 mM) hydrogel samples after exposure to trypsin (0.01% or 0.25% w/v) and TRIS over time at 37 °C. The degradation of BSA by trypsin enzymatic activity induces the release of FITC molecules conjugated to BSA fragments from the hydrogel matrix. The results show the effect of trypsin concentration and PEGDA concentration on the release kinetics of FITC. c) The Korsmeyer–Peppas theoretical model investigates drug release kinetics from cylindrical hydrogel systems. The release of FITC from our hydrogel is predominantly governed by diffusion and swelling mechanisms, as evident from the calculated n values within the range of *n* = 0.64 to *n* = 0.83 for cylindrical geometries. d) The impact of the shape‐morphing process on the FITC release rate was examined. The drug release rate was significantly increased when actuators underwent shape changes in the presence of trypsin at a physiological concentration of 0.01% w/v compared to passive hydrogels. However, at a higher trypsin concentration (0.25% w/v), there was no significant difference in FITC release rates between expandable bilayer and passive hydrogel samples. e) Close‐up images display the arc‐shaped, bilayer BSA(FITC)‐PEGDA hydrogel immersed in a trypsin solution (0.25% w/v, RT). These images capture the hydrogel's simultaneous shape‐morphing and FITC release processes, as evidenced by the diminishing fluorescent intensity within the BSA(FITC)‐PEGDA layer (Supporting Movie 2, Supporting Information).

To advance our system's capabilities beyond mere actuating behavior, we intended to integrate a controlled drug release mechanism to enhance efficiency and precision in targeted drug delivery significantly. Thus, the investigation extended into the bioconjugation of small molecules to BSA, with FITC selected as the representative drug model due to its facile attachment to proteins and its straightforward monitoring in solution (having excitation and emission wavelengths of 483 and 538 nm, respectively). This conjugation process entails creating covalent bonds between the isothiocyanate group of FITC and the amino groups located on the lysine residues within the BSA protein, resulting in BSA: FITC (BSA(FITC)) complexes (22:1 molar ratio) (**Figure** **4**ai,ii). Subsequently, these complexes were functionalized with PEGDA via aza‐Michael addition, yielding a BSA(FITC)‐PEGDA solution (Figure 4aiii), which was then combined with APS and TEMED to form a hydrogel structure of BSA(FITC)‐PEGDA (Figure 4aiv). A key finding of this phase was that the conjugation of FITC and PEGDA onto the BSA did not compromise the mechanical integrity or stiffness of the hydrogel in comparison to FITC‐free samples (Supporting Figure 5, Supporting Information).

After that, we investigated the release kinetics of FITC from these hydrogels under varying concentrations of trypsin. It was observed that an increase in trypsin concentration corresponded with a heightened release of FITC over time, indicating a controlled release facilitated by the enzymatic degradation of BSA within the hydrogel matrix. This phenomenon underlines the potential of enzyme‐triggered drug delivery mechanisms in hydrogel‐based systems (Figure 4b, Supporting Figure 9, Supporting Information).

Additionally, we delved into how alterations in the crosslinking density, manipulated through lysine functionalization with PEGDA, within the hydrogel network influenced the FITC release kinetics. The findings revealed that the degree of functionalization significantly affected the rate and total quantity of FITC released. Notably, hydrogels with 2–50 mM BSA‐PEGDA demonstrated a more pronounced FITC release than those with 2–100 mM BSA‐PEGDA (Figure 4b). This variability underscores the system's adaptability and highlights the importance of precise functionalization for tailored molecule release kinetics. Remarkably, hydrogels with a concentration of 2–50 mM BSA‐PEGDA were observed to dissolve entirely in a trypsin solution (0.25% w/v) within 48 h at 37 °C (Figure 4b, Supporting Figure 6, Supporting Information). Subsequently, at 72 h, BSA‐PEGDA hydrogels (2–100 mM) approached near‐complete release in the presence of trypsin (0.25%, w/v). At 120 h, BSA‐PEGDA hydrogels (2–50 mM) were completely dissolved in 0.01% trypsin. It should be noted that for BSA‐PEGDA hydrogels (2–100 mM) in 0.01% trypsin, the lack of reaching near‐complete release may be due to the deteriorating state of trypsin over time.^[^ ^70^ ^]^ Hydrogel samples in TRIS showed a slow release, which can be attributed to the PEGDA hydrolysis, which was approved by the complete hydrolysis of the hydrogel in TRIS at 37 °C after 45 days (Supporting Figure 6, Supporting Information).^[^ ^71^ ^]^

Thereafter, we applied the Korsmeyer–Peppas model to elucidate the release kinetics of FITC from the hydrogel carriers.^[^ ^72^, ^73^ ^]^ Our analysis revealed a linear relationship between the cumulative FITC release logarithm and time logarithm (Figure 4c). The corresponding n‐values for the FITC‐hydrogels in the presence of trypsin (0.01% and 0.25% w/v) were found to be greater than 0.45 but less than 0.89 for all BSA‐PEGDA (2–50, 2–100 mM) hydrogels (Supporting Table 1, Supporting Information). This suggests that the FITC release process was predominantly governed by diffusion and swelling or erosion of the hydrogel matrix resulting from the BSA digestion within the hydrogel matrices.

Furthermore, this study leveraged the preserved mechanical properties of the BSA(FITC)‐PEGDA hydrogels (Supporting Figure 5, Supporting Information) to develop a novel bilayer arc‐shaped, enzyme‐responsive drug release actuator. We used the 200 mM PEGDA as an outer layer and BSA(FITC)‐PEGDA (2–100 mM) as the inner layer. This cutting‐edge “active” actuator is distinct in its ability to integrate simultaneous shape morphing with controlled FITC release.

A key aspect of our research was investigating how changes in the actuator's shape could influence FITC release rates. To this end, we crafted an arc‐shaped actuator with a similar configuration but devoid of “passive” shape‐morphing capabilities (Supporting Figure 7, Supporting Information). Comparing the latter with the shape‐morphing actuator, especially when immersed in a solution containing a low physiological concentration of trypsin (0.01% w/v) over time at 37 °C, provided valuable insights. The findings underscored that the actuator's shape‐morphing characteristic significantly impacts the drug release rate (*p* < 0.01, Supporting Figure 8, Supporting Information). Notably, in the scenario with a higher enzyme concentration of trypsin (0.25% w/v), it was observed that the accelerated BSA degradation due to the high enzyme concentration effectively counterbalanced the shape‐induced effects on the release rate (*p* > 0.01) (Figure 4d, Supporting Figure 8). These findings significantly advance drug delivery systems, showcasing the potential of shape‐morphing hydrogels in controlled‐release applications (Figure 4e).

In the continuously improving field of stimuli‐responsive materials, hydrogel actuators are recognized as crucial tools in biomedical applications due to their ability to respond to environmental stimuli such as temperature and pH to drive targeted therapeutic actions. Hydrogel bilayers exploit the differential swelling properties to achieve precise bending or folding motions, which are useful in directed drug delivery and soft robotics.^[^ ^7^, ^74^, ^75^, ^76^, ^77^, ^78^, ^79^ ^]^ Microsized theragrippers employ shape‐memory polymers and enzyme‐biopolymer interactions to autonomously attach to biological tissue, enhancing drug delivery bioavailability.^[^ ^57^, ^80^, ^81^ ^]^ An enzyme‐triggered bilayer hydrogel actuator self‐folds upon collagenase exposure with gelatin methacrylate‐copolyethylene glycol dimethacrylate cleavage, reducing stiffness and enabling shape change.^[^ ^54^ ^]^ Additionally, 4D‐printed protein‐polymer hydrogels can undergo programmable shape‐morphing in response to temperature, pH, or enzymatic changes, such as trypsin.^[^ ^53^ ^]^ A recent study introduced a mechano‐controlled biocatalytic system using thrombin and its inhibitor hirudin within hybrid thrombin‐polymer hydrogel networks, regulating mechanical properties and inducing shape‐morphing behavior when stretched over 150%.^[^ ^55^ ^]^

While the latter existing studies have demonstrated the potential of enzyme‐triggered hydrogels in folding and gripping shape‐morphing behavior for various applications, our research introduces synergistic expandable enzyme‐triggered hybrid protein‐polymer hydrogel actuators with controlled FITC release under physiological trypsin concentrations (0.01% w/v), which may offer advantages in precise long‐residence drug delivery applications, particularly in dynamic biological environments like the GI. These hydrogels, composed of BSA and PEGDA, show a unique interplay of mechanical properties. PEGDA concentrations and BSA covalent integration correlate with stiffness, and BSA‐trypsin digestive interaction induces softening (Figure 1, 2). Our hydrogel's responsiveness is highly specific to proteases, such as trypsin, unlike other GI enzymes, such as lysozyme,^[^ ^82^ ^]^ which showed negligible interaction (Supporting Figure 10, Supporting Information). This specificity significantly reduces the risk of unintended activations, making our hydrogels suitable for precise biomedical applications in the small intestine. The controlled and fibrous microstructure could be relevant for tissue engineering applications where a material's rigidity and morphology may need to be modulated to match the surrounding tissue properties or to change during the healing process.^[^ ^83^, ^84^, ^85^, ^86^ ^]^ Furthermore, the concurrent increase in SR upon enzyme exposure further indicates the material's potential for delivering larger payloads in drug delivery applications, as the expanded matrix could accommodate and release therapeutic agents in a controlled manner.^[^ ^3^, ^72^, ^87^ ^]^

Cryo‐SEM analyses revealed post‐trypsin changes in the hydrogel's microstructure, like larger pore sizes and a controlled fibrous texture, coinciding with the observed mechanical and swelling alterations (Figure 2d). EDS confirmed these findings, with decreased sulfur content indicating BSA degradation (Figure 2e). The microstructural changes observed post‐trypsin treatment, such as developing a more fibrous mesh with increased pore size, are promising for tissue engineering.^[^ ^88^, ^89^, ^90^ ^]^ These alterations could facilitate cell infiltration and nutrient transport, which is critical for tissue regeneration. The enlarged pores mimic the extracellular matrix's structure, potentially providing a scaffold that better supports cell growth and differentiation.^[^ ^62^, ^91^ ^]^

The shape‐morphing behavior of our hydrogel actuators can be attributed to the differential swelling and stiffness between the hydrogel layers induced by enzymatic degradation. Trypsin selectively breaks down the BSA components, leading to uneven mechanical stress distribution and increased structure swelling. This dynamic action could create soft robotic systems that mimic natural movements, potentially revolutionizing minimally invasive surgery and targeted drug delivery by navigating and responding to the body's internal environment.

The release mechanism of FITC from our hydrogel aligns with the Kosmeyer–Peppas model, where diffusion and swelling processes predominate, as reflected by the n values ranging from 0.64 to 0.83 for cylindrical geometries. This indicates that the diffusion of FITC and the hydrogel matrix's adaptive response to swelling and enzymatic degradation are integral to the drug release kinetics. The observed increase in pore size and BSA digestion further demonstrates the hydrogel's dynamic adaptation to biological stimuli, highlighting a sophisticated interplay between its engineered structure and functionality within a biological setting.^[^ ^3^, ^72^ ^]^ The release of FITC in TRIS within the initial 24 h is minimal (less than 10%) compared to its release in trypsin solutions. Over time, however, there is a noticeable increase in FITC release, likely due to hydrolytic cleavage within the PEGDA hydrogels network. Predominantly, this process involves breaking ester bonds in the PEGDA network. Factors influencing this include the low molecular weight of PEGDA and the degree of crosslinking within the hydrogel structure (Supporting Figure 6, Supporting Information).^[^ ^71^ ^]^

This study uses BSA(FITC)‐PEGDA hydrogels to create a bilayer arc‐shaped actuator for enzyme‐responsive controlled release systems. It also investigates the significant role of shape‐morphing in release kinetics. When exposed to physiological concentrations of trypsin (0.01% w/v), the active actuator, which can change shape, displayed a faster release rate than the passive counterpart. This observation suggests the potential to control drug release rates by manipulating the hydrogel's shape (Figure 4, Supporting Figure 8). The enhanced release rate in the active actuator is due to the shape‐changing process speeding up the extrusion of FITC‐containing peptide fragments from the hydrogel's interior. Furthermore, stretching the inner hydrogel layer, which increases its contact area with the solution, also accelerates the degradation of BSA by trypsin. These findings provide a deeper understanding of how physical shape changes and biochemical interactions within the hydrogel can enhance drug release.

Recognizing the critical role of biocompatibility in biomedical applications, the cross‐linking reagents APS and TEMED, essential for forming our hydrogels, are consumed during the polymerization reaction (completed in under 10 min) and subsequently removed through several washes in TRIS. This process potentially improves the biocompatibility of our hydrogels.^[^ ^92^ ^]^ Nevertheless, we plan to conduct comprehensive biocompatibility studies, including cytotoxicity assays, hemocompatibility tests, and immunogenicity assessments, to validate our hydrogel's clinical suitability further. Supporting this approach, previous studies have shown similar redox reactions with reagents like PEGDA, APS, and TEMED to be nontoxic, biocompatible hydrogel scaffolds for tissue regeneration.^[^ ^93^, ^94^, ^95^ ^]^

However, our study is not without its limitations. The oxygen sensitivity of the hydrogel synthesis reaction can adversely affect the polymerization process and the precision in designing complex shapes. Moreover, our system's slow shape‐morphing response could limit its effectiveness in GI transit applications, where the transition time ranges from 2 to 8 h from ingestion to the small intestine.^[^ ^96^ ^]^

In our ongoing efforts, we aim to develop an enzyme‐triggered hybrid protein‐polymer actuator for the targeted release of therapeutic agents like doxorubicin. Building on current insights, we will investigate various BSA‐doxorubicin conjugate strategies,^[^ ^97^, ^98^, ^99^, ^100^ ^]^ aiming to enhance the efficacy of our enzyme‐triggered release mechanisms in cancer therapy. Furthermore, we plan to significantly improve the slow shape‐morphing response by applying protein engineering to modify protein structures and polymer properties. This strategy could enhance control over protein–enzyme catalytic interactions, quicken response times, and broaden applications in environments requiring rapid morphological adaptations.

Our research on enzyme‐responsive hybrid protein‐polymer hydrogels represents a noteworthy advancement in their application in biomedical and materials engineering. Overcoming limitations to improve reversible shape transformations will expand their use in drug delivery, adaptive wound dressings, and tissue engineering scaffolds. These hydrogels also hold promise for biosensing, offering rapid and targeted responses to biological signals. Future developments might incorporate these hydrogels into microfluidic devices, improving lab‐on‐a‐chip technologies and 3D‐printed soft robotics.

## 3 Experimental Section

3.1

3.1.1

##### Compressive Test

A mixture of BSA‐PEGDA or PEGDA solution was mixed with APS (1M) and TEMED (10 mM) in a volume ratio of 9:1:10. Then, this mixture was introduced into a cylindrical mold (9 mm diameter, 2.5 mm height) for 5 min at RT to form a hydrogel sample. These samples were washed three times in 5 mL TRIS for 5 min each using a rotating mixer, then equilibrated in 2 mL TRIS for 5 h at RT to ensure full swelling. Compression tests were conducted at RT at a 1 mm min^−1^ rate using an INSTRON mechanical tester (5000 N load cell). Young's modulus was calculated from the 0–10% slope of the stress‐strain curve. To evaluate the impact of cross‐linking components within the hydrogel, BSA‐PEGDA hydrogels (2–50, 2–75, and 2–100 mM) and PEGDA hydrogels (50, 75, and 100 mM) were prepared and soaked in TRIS buffer for 5 h to achieve full swelling. To assess the effect of enzyme concentrations on the hydrogel mechanical response, each BSA‐PEGDA (2–100 mM) hydrogel sample was immersed in 2 mL of trypsin solutions (0.01%, 0.1%, 0.25%, 0.5%, and 1% w/v, pH = 7.7) and incubated at 37 °C for 1, 6, 12, 24, and 48 h. Similarly, each PEGDA (100 mM) hydrogel was immersed in 2 mL of trypsin (0.25% w/v) and incubated at 37 °C for varying durations. The PEGDA (200 mM) hydrogel was also submerged in 2 mL of TRIS solution and maintained at 37 °C for different periods. Postincubation, samples were washed three times with 10 mL TRIS at RT on a rotating mixer and underwent compression testing. Based on this, the temperature variable, to assess the hydrogel's mechanical response, BSA‐PEGDA (2–100 mM) hydrogel samples were prepared and immersed in either 0.01% or 0.25% w/v trypsin or TRIS. These were stored at 4, 22, and 37 °C for 24 h. Postincubation, the hydrogels were washed with TRIS buffer and underwent compression testing. Each of the samples mentioned above was replicated three times.

##### SR

Cylindrical BSA‐PEGDA (2–100 mM) and PEGDA (100, 200 mM) hydrogel samples were prepared as described previously. BSA‐PEGDA samples were exposed to 0.01% and 0.25% w/v trypsin concentrations for 0, 1, 6, 12, 24, and 48 h at 37 °C. Control groups of BSA‐PEGDA (2–100 mM) and PEGDA (100, 200 mM) hydrogels were immersed in TRIS under similar conditions. Postexperiment, surface moisture was removed with absorbent paper, and hydrogels were weighed for initial wet weights (*W*_wet_). Each sample was washed three times with ddH_2_O at RT to eliminate TRIS residue, then frozen in liquid nitrogen for 5 min and lyophilized for 48 h. Each sample was repeated three times. Final dry weights (*W*_dry_) were measured, and the SR was calculated using the following equation:

(1) SR(%)=(Wwet−Wdry)Wdry×100

##### Cryo‐SEM

BSA‐PEGDA (2–100 mM) hydrogels were treated with 0.01% and 0.25% trypsin at 37 °C for 48 h alongside untreated controls of BSA‐PEGDA (2–100 mM) and PEGDA (100 mM) hydrogels. All samples were washed three times in 15 mL of ddH_2_O at RT for 30 min each. The hydrogels were freeze‐fixed between two 3 mm aluminum pans using a high‐pressure freezer (EM ICE, Leica). Subsequently, they were transferred under vacuum freezing conditions to a freeze‐fracture unit (EM ACE900, Leica) via a loading station (EM VCM, Leica). In the cryogenic chamber, samples were fractured with a cryogenic knife, etched at −100 °C for 10 min to sublimate surface ice, and sputter‐coated with 3 nm of carbon. Imaging was performed in an SEM chamber using an in‐lens secondary electron detector (Gemini SEM, Zeiss) at −120 °C, with pore area analyses conducted using ImageJ software.

##### Sulfur Element Distribution Analysis

EDS was used to analyze hydrogel samples’ sulfur elemental distribution. The samples were placed in a cryo‐SEM with a conductive surface suitable for vacuum conditions. The cryo‐SEM electron beams induced ionization and X‐ray emission from the samples, interacting with their atomic structure. The emitted X‐rays, indicative of specific elements, were captured by an energy‐dispersive spectrometer, generating a spectrum of X‐ray counts versus energy. Analysis of this spectrum with advanced software provided both qualitative and quantitative insights into the elemental composition of the samples.

##### Knots Shape Morphing Experiment

The hydrogel samples were prepared by syringe‐filling BSA‐PEGDA (2–100 mM) hydrogel mixture into 20 cm transparent polytetrafluoroethylene (PTFE) tubes (1 mm inner diameter). Following a 5 min polymerization, the gel was extruded into TRIS and washed three times for 5 min each. The hydrogel was then tied and shaped into a figure “8” and allowed to stabilize in TRIS. Shape morphing was observed by immersing one hydrogel sample in TRIS and another in 0.01% or 0.25% trypsin solutions at 4, 22, and 37 °C. Morphing processes were documented using a GoPro 10 camera, capturing a time‐lapse video at ten‐second intervals.

##### Bilayer Hydrogel Preparation and Shape‐Morphing Analysis

A bilayer hydrogel system was fabricated utilizing a two‐step molding process. Initially, the outer layer was formed by casting 200 mM PEGDA within a rectangular mold that measured 15 mm in length, 3 mm in width, and 1 mm in depth. Blue dye was incorporated into the PEGDA solution to enhance visibility during the experimental process. A lid was then placed over the mold to confine the solution, facilitating a consistent layer thickness of 1 mm. This assembly was allowed to rest at RT for 5 min to initiate polymerization. The PEGDA layer was gently rinsed with TRIS buffer to remove any unreacted monomers or excess dye. The layer was then carefully dried using absorbent paper to eliminate surface moisture. Once dried, the PEGDA layer was manually bent to conform to the curvature of the semicircular outer section of a secondary mold. Next, the inner layer was prepared using a BSA‐PEGDA mixture (2–100 mM). To visually distinguish this layer from the outer layer, red dye was introduced into the mixture. This red‐dyed BSA‐PEGDA solution was then carefully dispensed into the inner curvature created by the prebent blue PEGDA layer, ensuring that the thickness of this inner layer mirrored that of the outer layer at a 1:1 ratio. Following the assembly of the bilayer structure, it was once more subjected to a washing step and subsequently allowed to undergo swelling in TRIS to achieve equilibrium. The hydrogel's response to environmental stimuli was observed by immersing it in TRIS and trypsin solutions of two concentrations, 0.01% and 0.25% w/v, and incubating at 37 °C for 168 h. The bending angle (*θ*), representing the degree of deviation from the gel's initial arc shape, was meticulously recorded at various intervals to quantify the hydrogel's morphological changes over time. These measurements were captured using photographic imaging and analyzed with ImageJ software, precisely evaluating the hydrogel's straightening behavior in response to the different solution environments.

##### BSA‐FITC Bioconjugation

A carbonate buffer (100 mM, pH 8.2) was prepared by dissolving a carbonate capsule in 50 mL ddH_2_O and adjusting the pH to 8.2 with 1 M HCl. For FITC labeling, the BSA‐FITC (4.4–0.145 mM) mixture was stirred magnetically in the dark for 5 h and then centrifuged at 5000 rpm for 5 min to remove the foam. Then, the mixture underwent dialysis against fresh ddH_2_O, which was changed every 3 h for 24 h. Finally, it was rapidly frozen in liquid nitrogen and lyophilized for 48 h to produce FITC‐labeled BSA powder. BSA(FITC) (4.4 mM) was evenly dissolved in TRIS buffer. Subsequently, the FITC concentration was assessed using the standard calibration curve. The molar ratio of FITC to BSA was quantitatively established as 1:22.

##### FITC Cumulative Release

Cylindrical hydrogels were prepared using BSA (FITC)‐PEGDA (2–50, 2–100 mM), each with a volume of 150 μL (≈19.95 mg of BSA). These hydrogels underwent three consecutive wash cycles, involving immersion in 10 mL of TRIS for 30 min per cycle to remove unbound FITC components. After washing, the hydrogels were incubated in 3 mL trypsin solution (0.01% and 0.25% w/v) and TRIS. This incubation occurred at 37 °C with a shaking speed of 30 RPM to ensure consistent exposure to the enzyme. The experiment was designed to track the release kinetics of FITC from the hydrogels over 0 to 7 days. 100 μL samples are periodically taken from each hydrogel's surrounding solution for fluorescence testing at predetermined intervals. This testing assesses the amount of FITC released from the hydrogel. After each sampling, the removed volume is replaced with fresh trypsin solution to maintain consistent conditions. The release of FITC was quantified using a calibration curve, determining the total mass of FITC released into the surrounding 3 mL volume, denoted as *M*_t_. The percentage of cumulative FITC release from the hydrogels was then calculated using the formula:

(2) $$\text{Cumulative Release }\left(\%\right)=\frac{M_{t}\left(\text{mg}\right)}{M_{0}\left(\text{mg}\right)}\times 100$$

where *M*_0_ represents the initial mass of FITC in the hydrogel sample.

##### FITC Release Kinetics from Bilayer BSA‐PEGDA Actuator

Hydrogel samples were fabricated using a previously established arc‐shaped bilayer method. The active samples comprised an inner layer containing 32 μL of BSA(FITC)‐PEGDA. The passive samples, serving as controls, were cast using the same mold but without implementing the shape‐morphing feature of the active samples. To ensure consistency across experiments, the thickness of each hydrogel layer was meticulously measured to maintain an approximate dimension of 1 mm. Samples were then divided into two groups labeled passive and active, each immersed in various trypsin concentrations of 0.01% and 0.25% w/v in a total volume of 3 mL. These groups were incubated at a physiological temperature of 37 °C. The release of FITC from the hydrogels was closely monitored until near completion of the release process. To maintain consistency in sampling, the solution surrounding the hydrogel samples was homogenized by pipetting up and down 15 times before each measurement. This ensured a uniform distribution of FITC within the solution for accurate fluorescence assessment. After homogenization, 100 μL of the solution was carefully extracted for fluorescence testing. In tandem with this extraction, 100 μL of fresh corresponding trypsin solution was added to the sample to maintain the solution's volume and concentration. The cumulative release of FITC was then calculated using the methodology described earlier, which involves the quantitative analysis of FITC concentration via fluorescence against a calibration curve to determine the percentage of FITC released from the hydrogel samples over time.

## Conflict of Interest

The authors declare no conflict of interest.

## Author Contributions

**Luai R. Khour**: designed and conceived the research. **Yuchen Li**: performed the experiments, analyzed the data, and wrote the first draft of the manuscript. **Luai R. Khour**: wrote the final manuscript. All authors reviewed and approved the final manuscript.

## Supporting information

Supplementary Material

## Data Availability

The data that support the findings of this study are available from the corresponding author upon reasonable request.

## References

[1] N. A. Peppas , J. Z. Hilt , A. Khademhosseini , R. Langer , Adv. Mater. 2006, 18, 1345.

[2] K. Y. Lee , D. J. Mooney , Chem. Rev. 2001, 101, 1869. 11710233 10.1021/cr000108x

[3] J. Li , D. J. Mooney , Nat. Rev. Mater. 2016, 1, 16071. 29657852 10.1038/natrevmats.2016.71 PMC5898614

[4] S. Rittikulsittichai , A. G. Kolhatkar , S. Sarangi , M. A. Vorontsova , P. G. Vekilov , A. Brazdeikis , T. R. Lee , Nanoscale 2016, 8, 11851. 27227963 10.1039/c5nr09235c

[5] Z. H. Ghauri , A. Islam , M. A. Qadir , N. Gull , B. Haider , R. U. Khan , T. Riaz , Sci. Rep. 2021, 11, 21255. 34711866 10.1038/s41598-021-00452-x PMC8553746

[6] H. Haidari , Z. Kopecki , A. T. Sutton , S. Garg , A. J. Cowin , K. Vasilev , Antibiotics 2021, 10, 49. 33466534 10.3390/antibiotics10010049 PMC7824857

[7] Z. Han , P. Wang , G. Mao , T. Yin , D. Zhong , B. Yiming , X. Hu , Z. Jia , G. Nian , S. Qu , W. Yang , ACS Appl. Mater. Interfaces 2020, 12, 12010. 32053341 10.1021/acsami.9b21713

[8] S. Zhang , A. M. Bellinger , D. L. Glettig , R. Barman , Y. A. L. Lee , J. Zhu , C. Cleveland , V. A. Montgomery , L. Gu , L. D. Nash , D. J. Maitland , R. Langer , G. Traverso , Nat. Mater. 2015, 14, 1065. 26213897 10.1038/nmat4355 PMC4772966

[9] B. Maiti , A. Abramov , L. Franco , J. Puiggalí , H. Enshaei , C. Alemán , D. D. Díaz , Adv. Funct. Mater. 2020, 30, 2070150.

[10] J. J. Kang Derwent , W. F. Mieler , Trans. Am. Ophthalmol. Soc. 2008, 106, 206. 19277236 PMC2646442

[11] J. Li , Q. Ma , Y. Xu , M. Yang , Q. Wu , F. Wang , P. Sun , ACS Appl. Mater. Interfaces 2020, 12, 55290. 33232107 10.1021/acsami.0c17085

[12] M. Shahriari , M. Zahiri , K. Abnous , S. M. Taghdisi , M. Ramezani , M. Alibolandi , J. Control. Rel., 308, 2019. 10.1016/j.jconrel.2019.07.004 31295542

[13] M. Sobczak , Int. J. Mol. Sci. 23, 2022. 10.3390/ijms23084421 PMC9031066 35457239

[14] A. S. Hoffman , Adv. Drug Deliv. Rev. 2012, 64, 18.

[15] T. R. Hoare , D. S. Kohane , Polymer 2008, 49, 1993.

[16] J. L. Drury , D. J. Mooney , Biomaterials 2003, 24, 4337. 12922147 10.1016/s0142-9612(03)00340-5

[17] F. Xu , C. Dawson , M. Lamb , E. Mueller , E. Stefanek , M. Akbari , T. Hoare , Front. Bioeng. Biotechnol. 2022, 10, 849831. 35600900 10.3389/fbioe.2022.849831 PMC9119391

[18] K. Y. Lee , D. J. Mooney , Prog. Polym. Sci. 2012, 37, 106. 22125349 10.1016/j.progpolymsci.2011.06.003 PMC3223967

[19] P. Ray , M. Maity , H. Barik , G. S. Sahoo , M. S. Hasnain , M. N. Hoda , A. K. Nayak , Alginates Drug Delivery 2020, 41.

[20] J. Fu , F. Yang , Z. Guo , New J. Chem. 2018, 42, 17162.

[21] F. Ahmadi , Z. Oveisi , M. Samani , Z. Amoozgar , Res. Pharm. Sci. 2015, 10, 1. PMID: 26430453v. 26430453 PMC4578208

[22] J. R. Choi , K. W. Yong , J. Y. Choi , A. C. Cowie , Biotechniques 2019, 66, 40. 30730212 10.2144/btn-2018-0083

[23] K. McAvoy , D. Jones , R. R. S. Thakur , Pharm. Res. 2018, 35, 36. 29368249 10.1007/s11095-017-2298-9 PMC5784000

[24] M. A. Haq , Y. Su , D. Wang , Mater. Sci. Eng.: C 2017, 70, 842. 10.1016/j.msec.2016.09.081 27770962

[25] L. Tang , L. Wang , X. Yang , Y. Feng , Y. Li , W. Feng , Prog. Mater. Sci. 2021, 115, 100702.

[26] M. Khandaker , Int. J. Mater. Sci. 2013, 3, 133. 25478321 10.14355/ijmsci.2013.0304.01 PMC4251656

[27] N. Sheikh , L. Jalili , F. Anvari , Radiat. Phys. Chem. 2010, 79, 735.

[28] T. J. Deming , Adv. Mater. 1997, 9, 299.

[29] J. Gao , C. Tang , M. A. Elsawy , A. M. Smith , A. F. Miller , A. Saiani , Biomacromolecules 2017, 18, 826. 28068466 10.1021/acs.biomac.6b01693

[30] J. K. Wychowaniec , A. M. Smith , C. Ligorio , O. O. Mykhaylyk , A. F. Miller , A. Saiani , Biomacromolecules 2020, 21, 2285. 32275138 10.1021/acs.biomac.0c00229 PMC7304824

[31] E. F. Banwell , E. S. Abelardo , D. J. Adams , M. A. Birchall , A. Corrigan , A. M. Donald , M. Kirkland , L. C. Serpell , M. F. Butler , D. N. Woolfson , Nat. Mater. 2009, 8, 596. 19543314 10.1038/nmat2479 PMC2869032

[32] J. Fang , A. Mehlich , N. Koga , J. Huang , R. Koga , X. Gao , C. Hu , C. Jin , M. Rief , J. Kast , D. Baker , H. Li , Nat. Commun. 2013, 4, 2974. 24352111 10.1038/ncomms3974 PMC3983047

[33] C. P. Brown , M. D. G. Hughes , N. Mahmoudi , D. J. Brockwell , P. L. Coletta , S. Peyman , S. D. Evans , L. Dougan , Biomater. Sci. 2023, 11, 2726. 36815670 10.1039/d2bm01918c PMC10088474

[34] M. A. Da Silva , S. Lenton , M. Hughes , D. J. Brockwell , L. Dougan , Biomacromolecules 2017, 18, 636. 28006103 10.1021/acs.biomac.6b01877 PMC5348097

[35] M. D. G. Hughes , B. S. Hanson , S. Cussons , N. Mahmoudi , D. J. Brockwell , L. Dougan , ACS Nano 2021, 15, 11296. 34214394 10.1021/acsnano.1c00353 PMC8320229

[36] M. Slawinski , M. Kaeek , Y. Rajmiel , L. R. Khoury , Nano Lett. 2022, 22, 6942. 36018622 10.1021/acs.nanolett.2c01558 PMC9479135

[37] L. R. Khoury , J. Nowitzke , N. Dahal , K. Shmilovich , A. Eis , I. Popa , J. Visualized Exp. 2018, 138. 10.1007/10.3791/58280. PMC6231694 30199039

[38] L. R. Khoury , J. Nowitzke , K. Shmilovich , I. Popa , Macromolecules 2018, 51, 1441.

[39] L. R. Khoury , M. Slawinski , D. R. Collison , I. Popa , Sci. Adv. 2020, 6, 1. 10.1126/sciadv.aba6112 PMC7190360 32494690

[40] L. R. Khoury , I. Popa , Nat. Commun. 2019, 10, 5439. 31784506 10.1038/s41467-019-13312-0 PMC6884551

[41] Q. Bian , L. Fu , H. Li , Nat. Commun. 2022, 13, 137. 35013234 10.1038/s41467-021-27744-0 PMC8748998

[42] L. Fu , L. Li , Q. Bian , B. Xue , J. Jin , J. Li , Y. Cao , Q. Jiang , H. Li , Nature 2023, 618, 766. 10.1038/s41586-023-06037-0 37344650

[43] W. Sun , T. Duan , Y. Cao , H. Li , Biomacromolecules 2019, 20, 4199. 31553595 10.1021/acs.biomac.9b01114

[44] Y. Tang , X. Zhang , X. Li , C. Ma , X. Chu , L. Wang , W. Xu , Eur. Polym. J. 2022, 162, 110881.

[45] S. H. Kim , Y. K. Yeon , J. M. Lee , J. R. Chao , Y. J. Lee , Y. B. Seo , M. T. Sultan , O. J. Lee , J. S. Lee , S. Il Yoon , I. S. Hong , G. Khang , S. J. Lee , J. J. Yoo , C. H. Park , Nat. Commun. 2018, 9, 1620. 29693652 10.1038/s41467-018-03759-y PMC5915392

[46] J. Wu , P. Li , C. Dong , H. Jiang , B. Xue , X. Gao , M. Qin , W. Wang , B. Chen , Y. Cao , Nat. Commun. 2018, 9, 620. 29434258 10.1038/s41467-018-02917-6 PMC5809592

[47] E. Sanchez‐Rexach , P. T. Smith , A. Gomez‐Lopez , M. Fernandez , A. L. Cortajarena , H. Sardon , A. Nelson , ACS Appl. Mater. Interfaces 2021, 13, 19193. 33871260 10.1021/acsami.0c22377

[48] S. Yu , N. Sadaba , E. Sanchez‐Rexach , S. L. Hilburg , L. D. Pozzo , G. Altin‐Yavuzarslan , L. M. Liz‐Marzán , D. Jimenez de Aberasturi , H. Sardon , A. Nelson , Adv. Funct. Mater. 2023, 34, 2311209. 38966003 10.1002/adfm.202311209 PMC11221775

[49] M. Kaeek , L. R. Khoury , Adv. Sci. 2023, 10, 2306862. 10.1002/advs.202306862 PMC10754117 37991134

[50] Z. Li , Y. Zhou , T. Li , J. Zhang , H. Tian , View 2022, 3, 20200112.

[51] C. M. Wells , M. Harris , L. Choi , V. P. Murali , F. D. Guerra , J. A. Jennings , J. Funct. Biomater. 2019, 10, 34. 31370252 10.3390/jfb10030034 PMC6787590

[52] H. Wei , J. Cui , K. Lin , J. Xie , X. Wang , Bone Res. 2022, 10, 17. 35197462 10.1038/s41413-021-00180-y PMC8866424

[53] B. Narupai , P. T. Smith , A. Nelson , Adv. Funct. Mater. 2021, 31, 2011012.

[54] J. C. Athas , C. P. Nguyen , B. C. Zarket , A. Gargava , Z. Nie , S. R. Raghavan , ACS Appl. Mater. Interfaces 2016, 8, 19066. 27404225 10.1021/acsami.6b05024

[55] K. Zhang , Y. Zhou , J. Zhang , Q. Liu , C. Hanenberg , A. Mourran , X. Wang , X. Gao , Y. Cao , A. Herrmann , L. Zheng , Nat. Commun. 2024, 15, 249. 38172560 10.1038/s41467-023-44607-y PMC10764310

[56] K. Moriyama , S. Nakao , M. Tsuji , N. Nakagawa , T. Satake , Y. Johno , Biochem. Eng. J. 2024, 208, 109338.

[57] N. Bassik , A. Brafman , A. M. Zarafshar , M. Jamal , D. Luvsanjav , F. M. Selaru , D. H. Gracias , J. Am. Chem. Soc. 2010, 132, 16314. 20849106 10.1021/ja106218s PMC2988106

[58] I. Sensoy , Curr. Res. Food Sci. 2021, 4, 308. 34027433 10.1016/j.crfs.2021.04.004 PMC8134715

[59] W. H. Godfrey , M. D. Kornberg , Metabolites 2020, 10, 426. 33114536 10.3390/metabo10110426 PMC7693344

[60] Y. Liu , D. Zhang , Z.‐Y. Qiao , G.‐B. Qi , X.‐J. Liang , X.‐G. Chen , H. Wang , Y. Liu , X. Chen , D. Zhang , Z. Qiao , G. Qi , X. Liang , H. Wang , Adv. Mater. 2015, 27, 5034. 26198072 10.1002/adma.201501502

[61] S. L. Buffington , J. E. Paul , M. M. Ali , M. M. Macios , P. T. Mather , J. H. Henderson , Acta Biomater. 2019, 84, 88. 30471473 10.1016/j.actbio.2018.11.031

[62] Y. Efraim , B. Schoen , S. Zahran , T. Davidov , G. Vasilyev , L. Baruch , E. Zussman , M. Machluf , Sci. Rep. 2019, 9, 5578. 30944384 10.1038/s41598-019-41831-9 PMC6447624

[63] D. Li , H. Zhang , Eur. J. Chem. 2014, 5, 287.

[64] M. Bhattacharya , N. Jain , K. Bhasne , V. Kumari , S. Mukhopadhyay , J. Fluoresc. 2011, 21, 1083. 21128099 10.1007/s10895-010-0781-3

[65] N. El Kadi , N. Taulier , J. Y. Le Huérou , M. Gindre , W. Urbach , I. Nwigwe , P. C. Kahn , M. Waks , Biophys. J. 2006, 91, 3397. 16861279 10.1529/biophysj.106.088963 PMC1614494

[66] T. Peters , Adv. Protein Chem. 1985, 37, 161. 3904348 10.1016/s0065-3233(08)60065-0

[67] I. Piovarci , S. Melikishvili , M. Tatarko , T. Hianik , M. Thompson , Biosensors 2021, 11, 117. 33920444 10.3390/bios11040117 PMC8070231

[68] A. Bougatef , R. Balti , R. Nasri , K. Jellouli , N. Souissi , M. Nasri , J. Agric. Food Chem. 2010, 58, 5763. 20405833 10.1021/jf100534a

[69] C. W. V. dos Santos , M. E. da Costa Marques , H. de Araújo Tenório , E. C. de Miranda , H. J. Vieira Pereira , Biochem. Biophys. Rep. 2016, 8, 29. 28955938 10.1016/j.bbrep.2016.08.003 PMC5613698

[70] M. C. Ferrall‐Fairbanks , C. A. Kieslich , M. O. Platt , Proc. Natl. Acad. Sci. USA 2020, 117, 3307. 31980525 10.1073/pnas.1912207117 PMC7022172

[71] M. B. Browning , S. N. Cereceres , P. T. Luong , E. M. Cosgriff‐Hernandez , J. Biomed. Mater. Res A 2014, 102, 4244. 24464985 10.1002/jbm.a.35096 PMC4112173

[72] P. Colombo , R. Bettini , P. Santi , A. De Ascentiis , N. A. Peppas , J. Controlled Release 1996, 39, 231.

[73] M. E. Rabeh , L. K. Vora , J. V. Moore , M. F. Bayan , C. P. McCoy , M. P. Wylie , Biomater. Adv. 2024, 157, 213735. 38154402 10.1016/j.bioadv.2023.213735

[74] H. W. Huang , A. J. Petruska , M. S. Sakar , M. Skoura , F. Ullrich , Q. Zhangm , S. Pane , B. J. Nelson , in Proc. of the Annual Int. Conf. of the IEEE Engineering in Medicine and Biology Society, EMBC, USA 2016, p. 2103. 10.1109/EMBC.2016.7591143 28268746

[75] D. I. Kim , H. Lee , S. H. Kwon , Y. J. Sung , W. K. Song , S. Park , Adv. Healthcare Mater. 2020, 9, 2000118. 10.1002/adhm.202000118 32431072

[76] S. Miar , C. A. Perez , J. L. Ong , T. Guda , J. Biomater. Appl. 2019, 34, 523. 31291789 10.1177/0885328219861614 PMC6782056

[77] N. Bassik , B. T. Abebe , K. E. Laflin , D. H. Gracias , Polymer 2010, 51, 6093.

[78] K. Baek , J. H. Jeong , A. Shkumatov , R. Bashir , H. Kong , Adv. Mater. 2013, 25, 5568. 23864483 10.1002/adma.201300951

[79] A. Pantula , B. Datta , Y. Shi , M. Wang , J. Liu , S. Deng , N. J. Cowan , T. D. Nguyen , D. H. Gracias , Sci. Rob. 2022, 7, 1. 10.1126/scirobotics.add2903 36516274

[80] K. Malachowski , J. Breger , H. R. Kwag , M. O. Wang , J. P. Fisher , F. M. Selaru , D. H. Gracias , Angew. Chem. Int. Ed. Engl. 2014, 53, 8045. 24634136 10.1002/anie.201311047 PMC4315180

[81] A. Ghosh , L. Li , L. Xu , R. P. Dash , N. Gupta , J. Lam , Q. Jin , V. Akshintala , G. Pahapale , W. Liu , A. Sarkar , R. Rais , D. H. Gracias , F. M. Selaru , Sci. Adv. 2020, 6, 4133. 10.1126/sciadv.abb4133 PMC7608789 33115736

[82] J. Hankiewicz , E. Swierczek , Clin. Chim. Acta 1974, 57, 205. 4434640 10.1016/0009-8981(74)90398-2

[83] B. Yi , Q. Xu , W. Liu , Bioact. Mater. 2022, 15, 82. 35386347 10.1016/j.bioactmat.2021.12.005 PMC8940767

[84] X. Lu , Z. Ding , F. Xu , Q. Lu , D. L. Kaplan , ACS Appl. Bio Mater. 2019, 2, 3108. 10.1021/acsabm.9b00445 35030802

[85] G. Chen , C. Dong , L. Yang , Y. Lv , ACS Appl. Mater. Interfaces 2015, 7, 15790. 26151287 10.1021/acsami.5b02662

[86] T. Luo , B. Tan , L. Zhu , Y. Wang , J. Liao , Front. Bioeng. Biotechnol. 2022, 10, 817391. 35145958 10.3389/fbioe.2022.817391 PMC8822157

[87] P. Pal , J. P. Pandey , G. Sen , Int. J. Biol. Macromol. 2018, 113, 1116. 29505871 10.1016/j.ijbiomac.2018.02.143

[88] N. Latifi , M. Asgari , H. Vali , L. Mongeau , Sci. Rep. 2018, 8, 1047. 29348423 10.1038/s41598-017-18523-3 PMC5773686

[89] D. L. Matera , K. M. DiLillo , M. R. Smith , C. D. Davidson , R. Parikh , M. Said , C. A. Wilke , I. M. Lombaert , K. B. Arnold , B. B. Moore , B. M. Baker , Sci. Adv. 2020, 6, 1. 10.1126/sciadv.abb5069 PMC11206459 32917680

[90] Y. Chen , Y. Hao , A. Mensah , P. Lv , Q. Wei , Biomater. Adv. 2022, 136, 212799. 35929334 10.1016/j.bioadv.2022.212799

[91] I. Jun , H. S. Han , J. R. Edwards , H. Jeon , Int. J. Mol. Sci. 2018, 19, 745. 29509688 10.3390/ijms19030745 PMC5877606

[92] E. S. Desai , M. Y. Tang , A. E. Ross , R. A. Gemeinhart , Biomed. Mater. 2012, 7, 024108. 22455976 10.1088/1748-6041/7/2/024108 PMC3358450

[93] B. Göppert , T. Sollich , P. Abaffy , A. Cecilia , J. Heckmann , A. Neeb , A. Bäcker , T. Baumbach , F. J. Gruhl , A. C. B. Cato , Small 2016, 12, 3985. 27240250 10.1002/smll.201600683

[94] Y. Hwang , N. Sangaj , S. Varghese , Tissue Eng. Part A 2010, 16, 3033. 20486791 10.1089/ten.TEA.2010.0045

[95] H. D. Kim , J. Kim , R. H. Koh , J. Shim , J. C. Lee , T. Il Kim , N. S. Hwang , ACS Biomater. Sci. Eng. 2017, 3, 2470. 33445304 10.1021/acsbiomaterials.7b00299

[96] H. Minami , R. W. Mccallum , Gastroenterology 1984, 86, 1592. 6370777

[97] F. E. Chamberlain , R. L. Jones , S. P. Chawla , Future Oncol. 2019, 15, 1429. 30873850 10.2217/fon-2018-0922

[98] C. Du , D. Deng , L. Shan , S. Wan , J. Cao , J. Tian , S. Achilefu , Y. Gu , Biomaterials 2013, 34, 3087. 23374705 10.1016/j.biomaterials.2013.01.041

[99] F. Kratz , J. Drevs , G. Bing , C. Stockmar , K. Scheuermann , P. Lazar , C. Unger , Bioorg. Med. Chem. Lett. 2001, 11, 2001. 11454467 10.1016/s0960-894x(01)00354-7

[100] H. Liu , M. Sun , Z. Liu , C. Kong , W. Kong , J. Ye , J. Gong , D. C. S. Huang , F. Qian , J. Controlled Release 2019, 296, 40. 10.1016/j.jconrel.2019.01.014 30653981

