# Supplementary material for: Design and Functionality of Trypsin‐Triggered, Expandable Bovine Serum Albumin‐Polyethylene Glycol Diacrylate Hydrogel Actuators
Source: Small Sci. 2024 Jul 21;4(10):2400214. doi: 10.1002/smsc.202400214 (PMC11935001; doi:10.1002/smsc.202400214)
Supplement: Supplementary file 1 — Supplementary Material [file SMSC-4-2400214-s001.zip › smsc.202400214-sup-0001-suppdata-S1.pdf]

Supporting Information

Trypsin-Triggered Hybrid BSA-PEGDA Hydrogel Actuator for Drug Delivery

Yuchen LIU [1], *Luai R. Khoury* [1*]

[1] Department of Materials Science and Engineering, Technion Israel Institute of Technology, Haifa, 32000, Israel

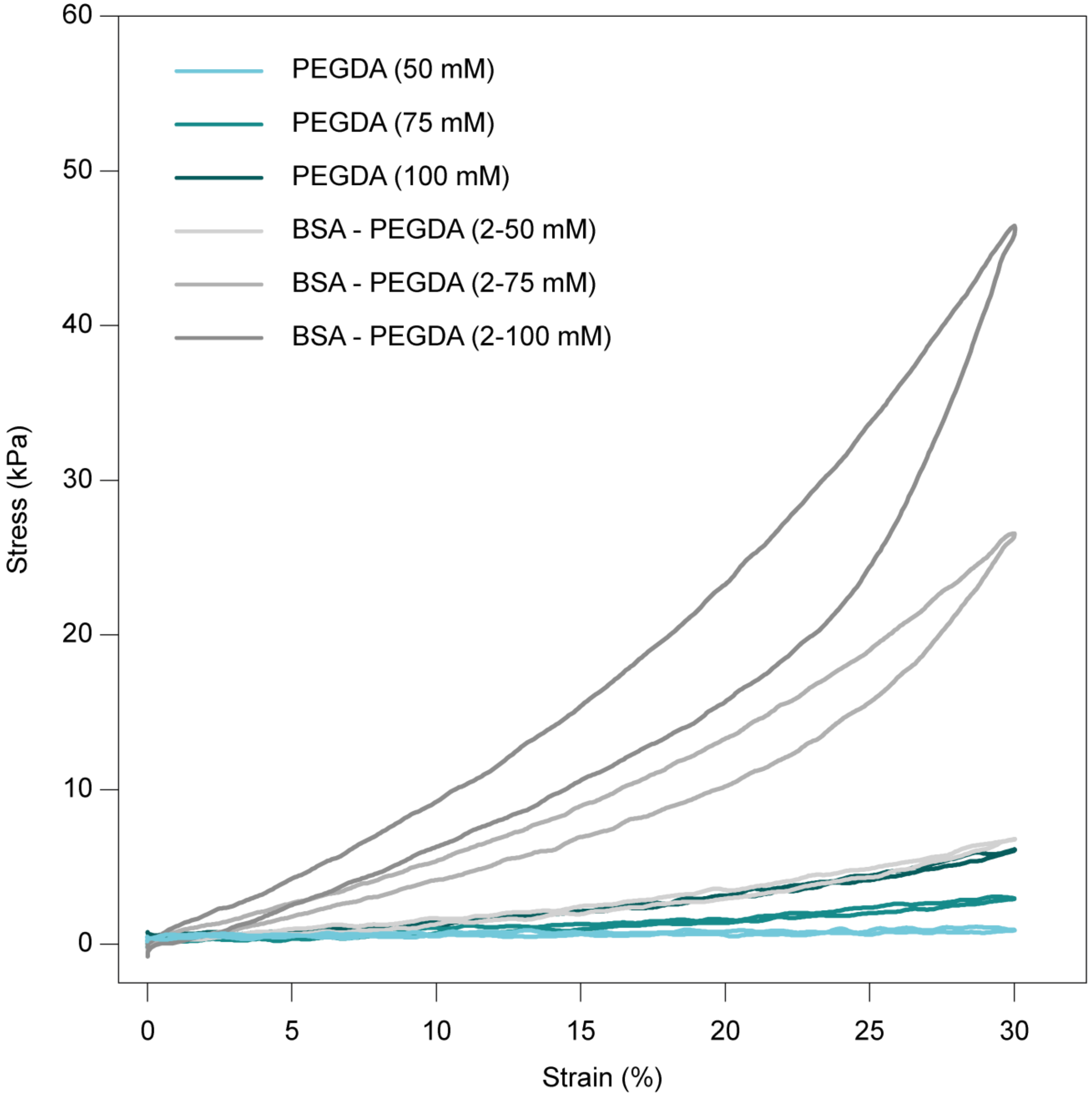

Supporting Figure 1. Comparative mechanical analysis of BSA-PEGDA (2-50, 2-75, and 2-100 mM) and PEGDA (50, 75, and 100 mM) hydrogels in TRIS buffer. The graph represents stress-strain responses for both BSA-PEGDA and PEGDA hydrogels immersed in TRIS, subjected to compression up

to 30% of their original height at a constant rate of 1 mm/min. It highlights how hydrogel stiffness is significantly affected by varying PEGDA and BSA concentrations. Notably, an increase in BSA from 0 mM to 2 mM and PEGDA from 50 mM to 100 mM markedly enhances hydrogel stiffness.

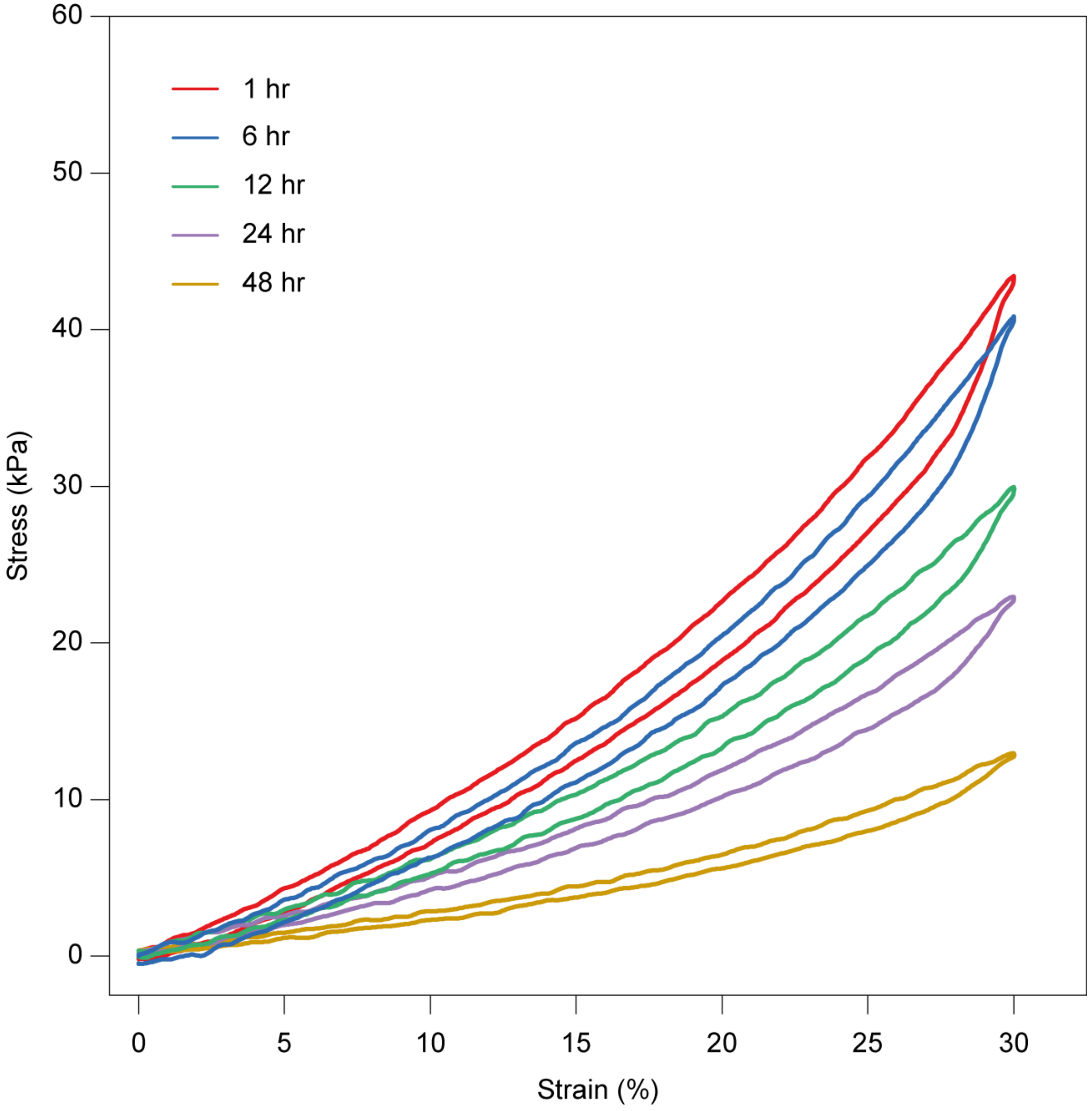

Supporting Figure 2. Mechanical behavior of BSA-PEGDA (2-100 mM) hydrogels in trypsin solution over time. The stress-strain curves depict the mechanical response of BSA-PEGDA hydrogels subjected to compression until reaching 30% of their initial height at a controlled rate of 1 mm/min, while immersed in a 0.01% w/v trypsin solution at 37°C. Over the duration of exposure to trypsin, a progressive decline in

hydrogel stiffness was observed, indicative of enzymatic degradation impacting the hydrogel's structural integrity.

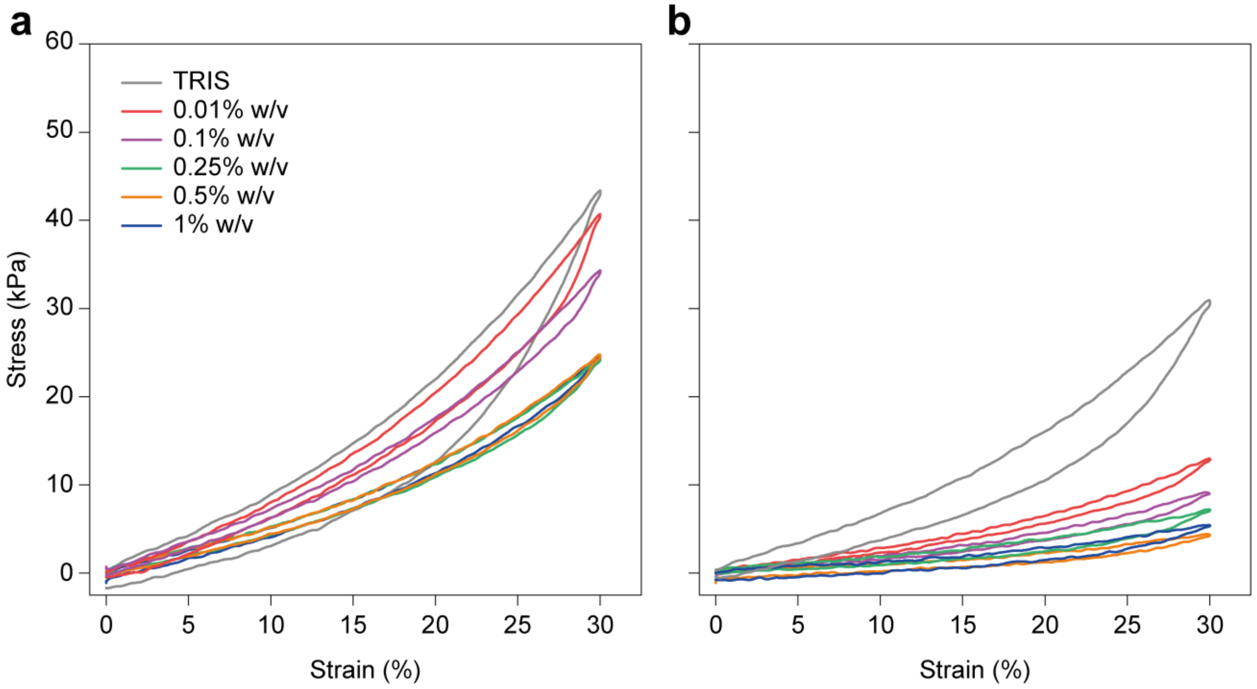

Supporting Figure 3. Mechanical characterization of BSA-PEGDA (2-100 mM) hydrogels after exposure to trypsin in varying concentrations and TRIS buffer. Stress-strain measurements were performed on BSA-PEGDA hydrogels subjected to 30% compression in 1 mm/min-controlled rate, post-immersion in different trypsin concentrations within TRIS at 37°C for intervals of (a) 6 and (b) 48 hours. The data reveal a time-dependent and concentration-dependent decrease in hydrogel stiffness, with elevated trypsin levels accelerating the stiffness decrease of the hydrogel structure.

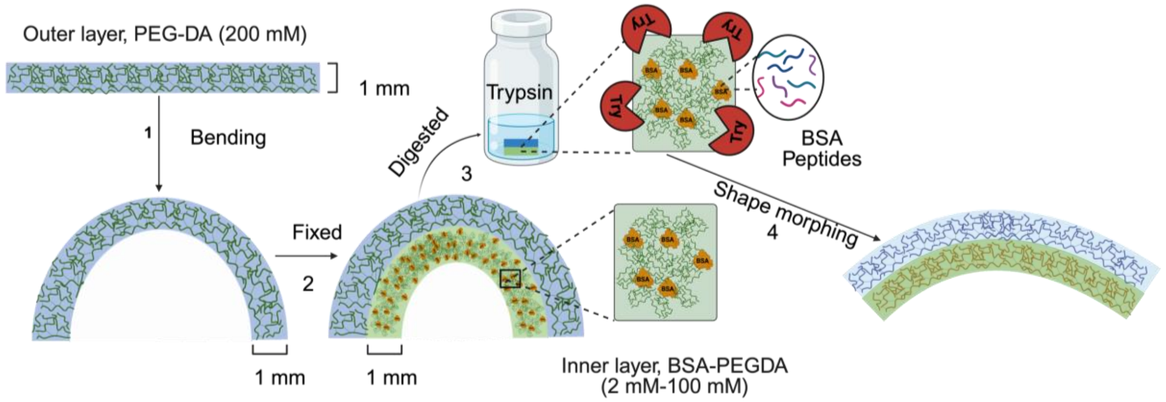

Supporting Figure 4. Fabrication of a responsive double-layer hydrogel system. The process begins with forming a 1 mm thick PEGDA (200 mM) outer layer in a rectangular mold, which is then molded into a curved shape within a semicircular mold. BSA-PEGDA (2-100 mM) or BSA (FITC)-PEGDA (2-100 mM) mixtures are subsequently applied to the inner layer to set the hydrogel into an arc configuration. Upon immersion in trypsin solution, the BSA-PEGDA inner layer is selectively degraded, causing a decrease in its mechanical strength and increasing its swelling ratio. This selective degradation enables the more stable outer PEGDA layer to attempt to revert to its initial flat shape. The dynamic interplay between the degrading inner layer and the resilient outer layer induces the hydrogel to transition gradually from a curved arc to a straight configuration.

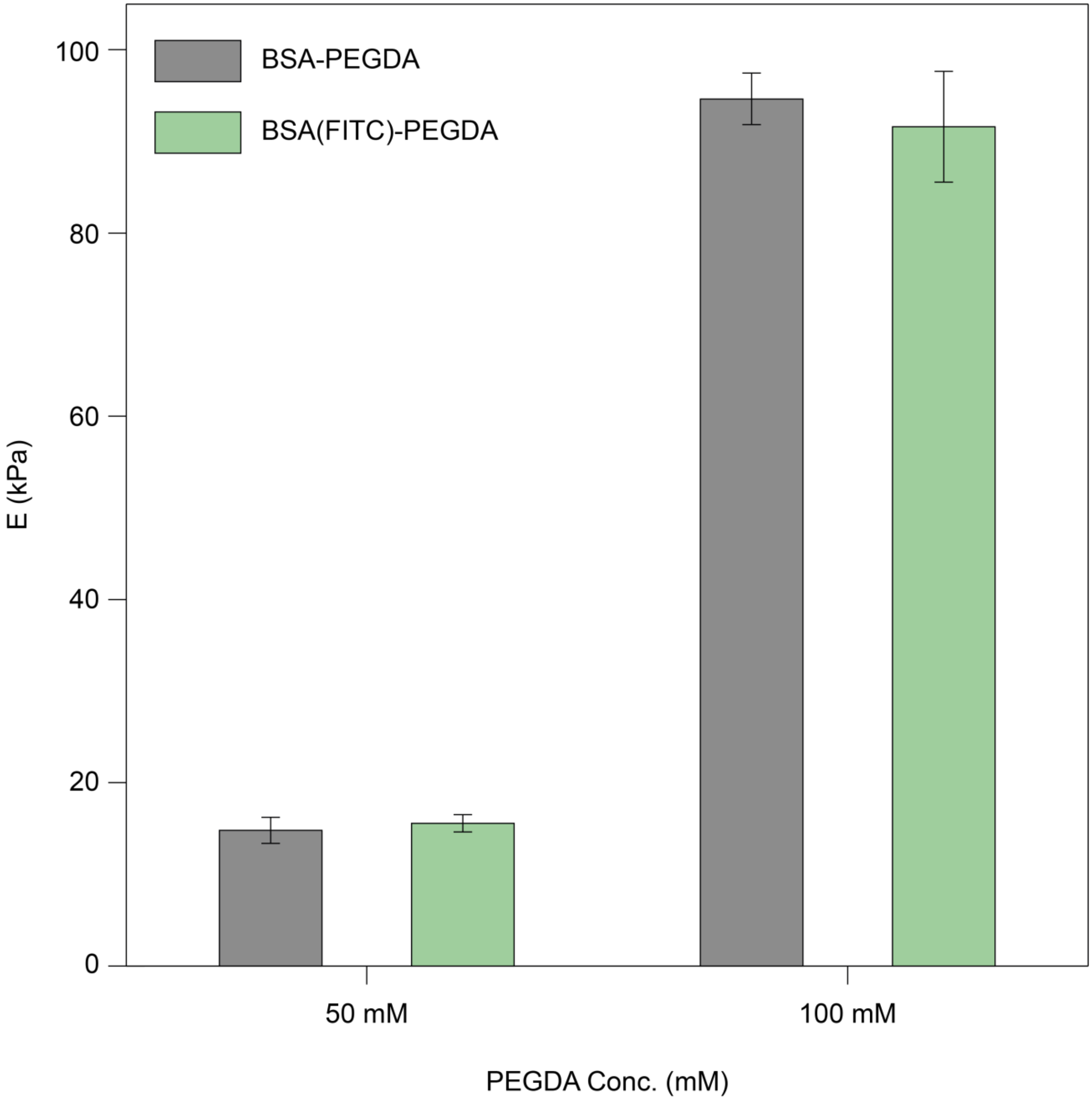

Supporting Figure 5. Assessing the impact of BSA-FITC conjugation on hydrogel stiffness. Comparative mechanical analysis was conducted on hydrogels formulated with 2-50 mM and 2-100 mM FITC-labeled BSA (BSA-FITC)-PEGDA versus non-labeled BSA-PEGDA hydrogels. The data suggests that FITC conjugation to BSA does not significantly influence the stiffness of the hydrogel samples, thereby maintaining the mechanical integrity of the hydrogel matrix after the labeling process.

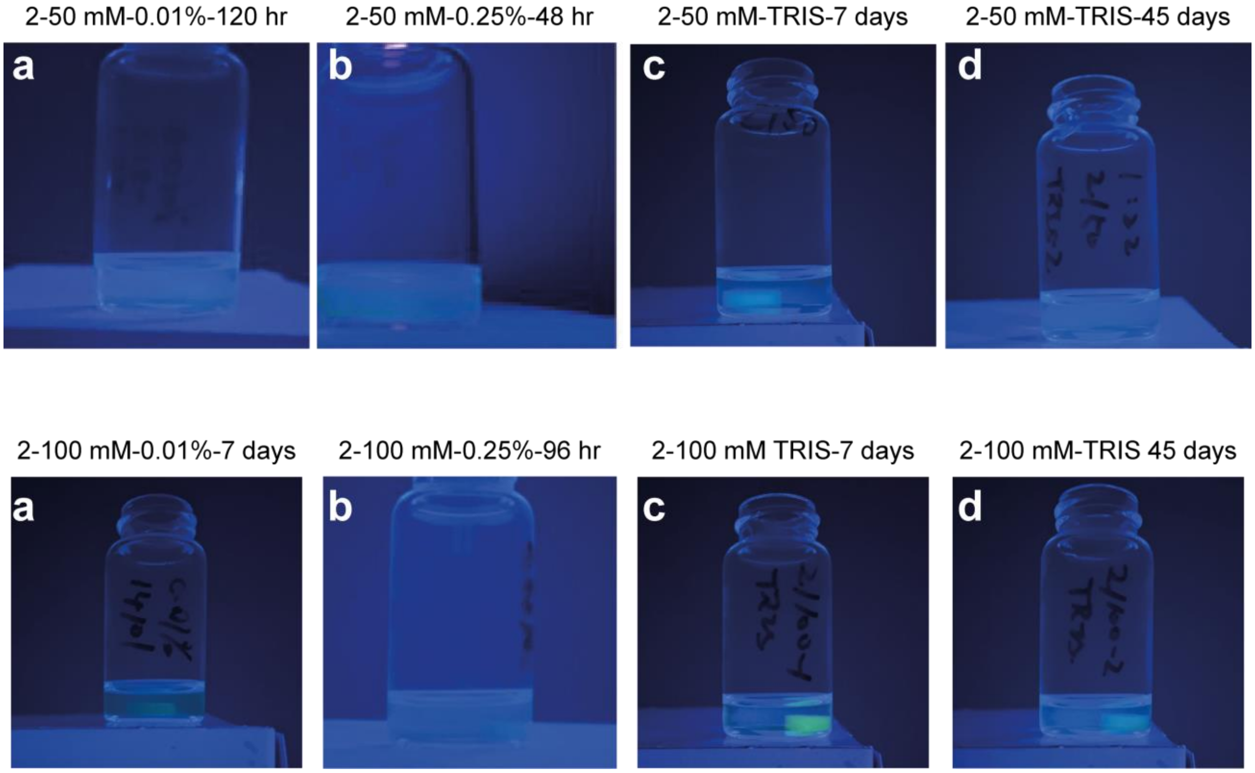

Supporting Figure 6. Examination of BSA-PEGDA hydrogel degradation post-FITC release. Topline: (a,b) After incubation at 37 °C, the hydrogel (2-50 mM) samples were fully degraded following exposure to 0.01% and 0.25% w/v trypsin solutions for 120 and 48 hours, respectively. (c) At 37°C, the hydrogel (2-50 mM) immersed in TRIS for up to 7 days exhibited green fluorescence under UV light. (d) The BSA-PEGDA (2-50 mM) hydrogel was completely degraded in TRIS buffer after a prolonged period of 45 days. Bottomline: (a) At 37°C, the hydrogel (2-100 mM) immersed in 0.01% trypsin showed faint green fluorescence under UV light after 7 days, indicating that it had not completely released FITC. (b) The hydrogel (2-100 mM) did not exhibit significant green fluorescence characteristics under UV light but still retained its three-dimensional gel framework. This indicates that the hydrogel exhibited complete release of FITC in a 0.25% w/v trypsin solution within 96 hours. (c) At 37°C, the hydrogel (2-100 mM) immersed in TRIS for up to 7 days exhibited strong green fluorescence under UV light, and in combination with observation (d) that the hydrogel (2-100 mM) still showed fluorescence after 45 days of immersion in TRIS under UV light, it indicates that hydrogels with high crosslinking network density are not easily hydrolyzed naturally. This can better reduce the release of drugs under non-specific stimuli.

BSA - PEGDA	Korsmeyer - Peppas (R^2)	n	K_m
2-50 mM, 0.25% w/v	0.99	0.74	9.03
2-100 mM, 0.25% w/v	0.99	0.73	5.76
2-50 mM, 0.01% w/v	0.98	0.64	4.52
2-100 mM, 0.01% w/v	0.99	0.83	1.6
2-50 mM, TRIS	0.98	0.69	1.08
2-100 mM, TRIS	0.99	0.74	0.55

Supporting Table 1. Analysis of diffusion and degradation-controlled release in FITC-labeled hydrogels using the Korsmeyer-Peppas model. The calculated 'n-values' for 2-50 mM and 2-100 mM FITC-hydrogels in the presence of 0.01% and 0.25% w/v trypsin, as well as in TRIS buffer, suggest the release mechanisms are governed by a combination of diffusion processes and enzymatic degradation.

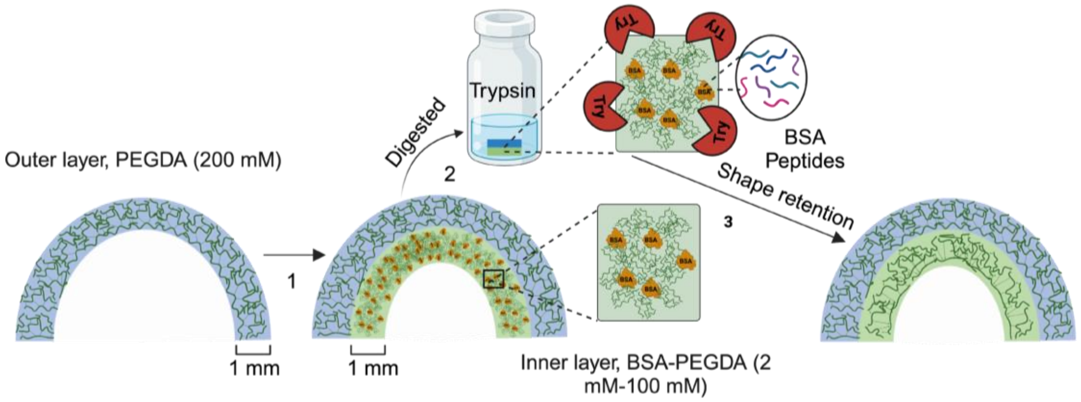

Supporting Figure 7. Fabrication of a passive arc-shaped hydrogel structure. The procedure starts by crafting an arc-shaped hydrogel layer using 200 mM PEGDA. A pre-gel BSA (FITC)-PEGDA solution at 2-100 mM concentration was deposited in the inner layer, creating a bilayer arc-shaped hydrogel. Upon immersion in trypsin, the BSA component of the inner layer was enzymatically degraded, diminishing its stiffness and increasing its swelling ratio. Despite these internal changes, the overall shape of the hydrogel remained unchanged, highlighting the passive nature of the arc-shaped structure, which, unlike active hydrogels, does not undergo significant morphological transformations in response to enzymatic stimuli.

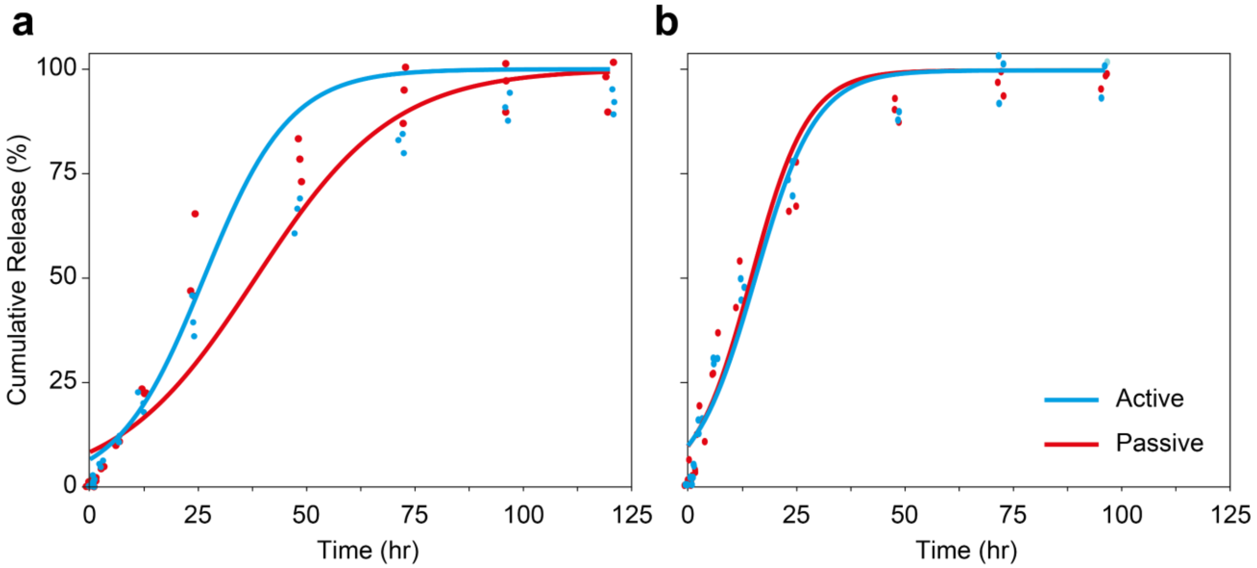

Supporting Figure 8. Comparative Dynamics of Cumulative Release in Active vs. Passive Hydrogels. (a) Logistic growth analysis revealed distinct cumulative release patterns between Active and Passive hydrogel groups, demonstrating a more rapid release rate in the Active group with a combined rate of k1 + k2 = 0.10, as opposed to k1 = 0.06 in the Passive group. The Active group reached the inflection point, signaling a slowdown in release, at 26.27 hours, earlier than the Passive group's 38.23 hours. These results, supported by a high model fit (R^2 = 0.967), highlight significant temporal differences in release dynamics between the groups (Supporting Table 2). (b) The Passive group exhibited a steady growth rate (k1 = 0.15) with a statistically significant inflection point at 14.54 hours. In contrast, the Active group's adjusted growth rate (k2 = -0.01) and inflection point (x02 = 0.92) did not significantly diverge from the Passive, suggesting similar growth trends (Supporting Table 3).

Supporting Table 2. Summary of Logistic Growth Model for Value by Time and Group (N = 66)

	**value**		
Predictors	*Estimates*	*CI*	*p*
Base growth rate – Passive (k1)	0.06	0.05 – 0.07	<0.001
Adjustment to the growth rate – Active (k2)	0.04	0.02 – 0.06	0.001
Base inflection point – Passive (x01)	38.23	34.56 – 41.91	<0.001
Adjustment to the inflection point – Active (x02)	-11.96	-16.61 – -7.30	<0.001

Notes: R^2 = 0.967; baseline group: passive

Supporting Table 3. Summary of Logistic Growth Model for Value by Time and Group (N = 60)

Predictors	*Estimates*	*CI*	*p*
Base growth rate – Passive (k1)	0.15	0.12 – 0.18	<0.001
Adjustment to the growth rate – Active (k2)	-0.01	-0.05 – 0.03	0.638
Base inflection point – Passive (x01)	14.54	12.73 – 16.35	<0.001
Adjustment to the inflection point – Active (x02)	0.92	-1.71 – 3.55	0.485

Notes: R^2 *= 0.961; baseline group: passive*

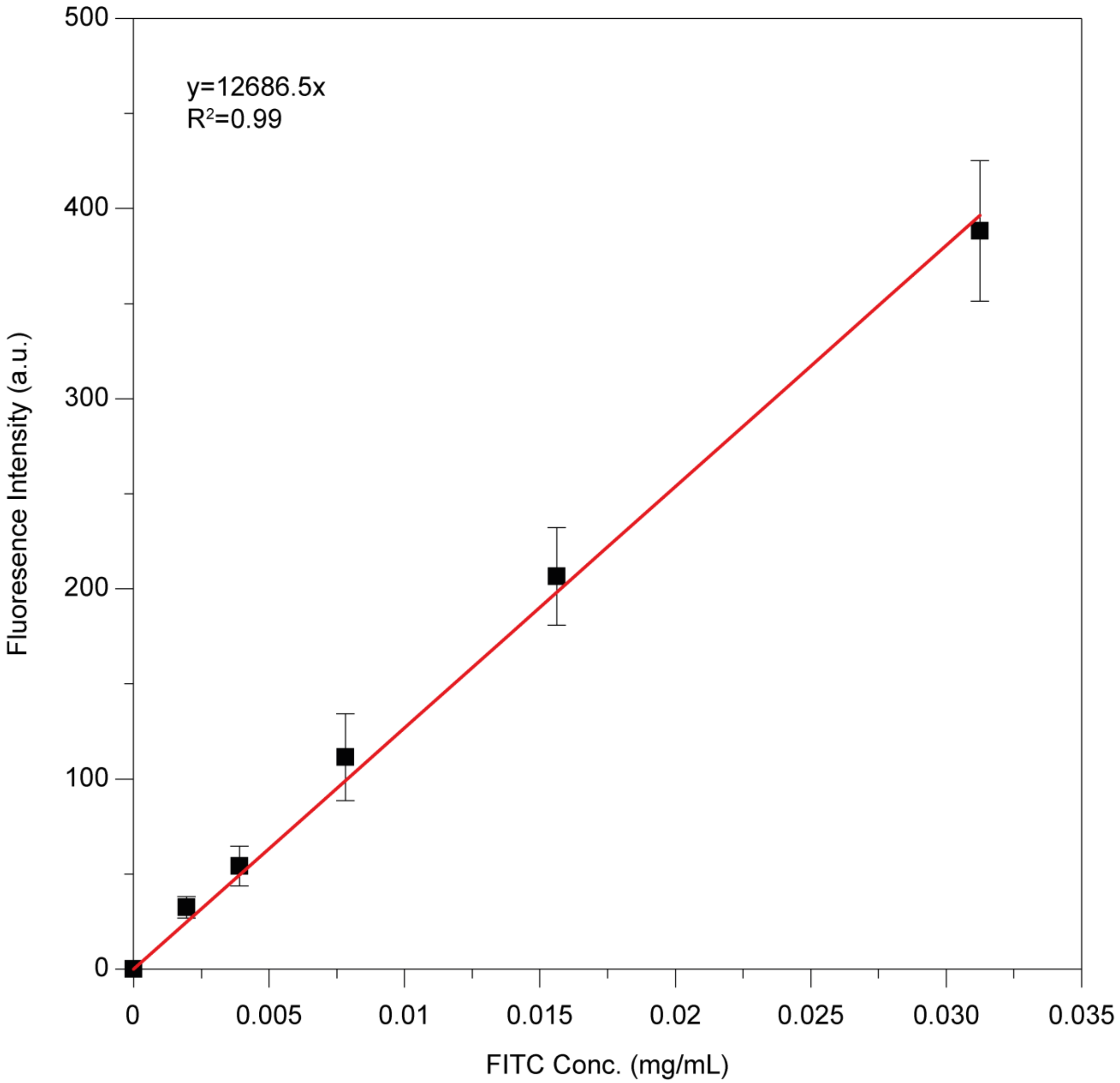

Supporting Figure 7. The calibration curve of FITC. FITC stock solution was prepared at a concentration of 0.642 mM in a total volume of 1 mL. From this stock solution, 200 μL was carefully drawn and aliquoted into two separate portions, each containing 100 μL. One portion was designated for direct fluorescence testing, while the other served as the initial dilution sample. The dilution process was performed by combining 100 μL of the FITC solution with 100 μL of TRIS buffer, and this step was methodically repeated eight times to achieve a serial dilution series. Following the preparation of dilutions, fluorescence intensity readings were taken using 100 μL from the final dilution. The measurements were conducted at an excitation wavelength of 483 nm and an emission wavelength of 538 nm. The measurements established a calibration curve correlating FITC concentration with fluorescence intensity. Each sample was repeated three times.

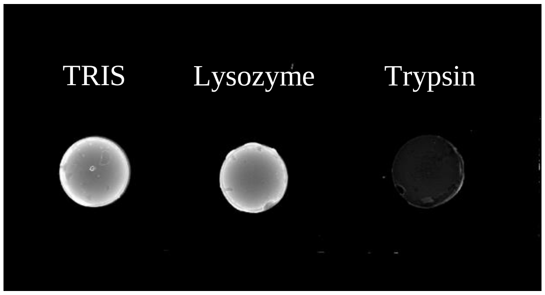

Supporting Figure 10. Specificity and Sensitivity of BSA-PEGDA Hydrogels to Trypsin Enzymatic Activity: Comparative Fluorescence Imaging Post-Treatment. BSA-PEGDA hydrogels (2 – 100 mM) were treated with TRIS, 0.01% w/v lysozyme, and 0.01% w/v trypsin at 37°C for 5 days to evaluate their responsiveness to trypsin enzymatic activity. Post-treatment, each hydrogel sample underwent a thorough washing process consisting of three 30-minute washes in TRIS at room temperature to eliminate residual enzymes and soluble degradation products. After washing, the hydrogels were immersed in TRIS containing 8-Anilinonaphthalene-1-sulfonic acid (ANS), a dye that binds to exposed hydrophobic protein sites, enhancing fluorescence. This property makes ANS an excellent tool for detecting protein structure or exposure alterations. Fluorescence imaging under UV light using a ChemDoc system indicated notable differences in response: hydrogels exposed to lysozyme and TRIS alone displayed similar fluorescence levels, suggesting that lysozyme had minimal impact on the hydrogel's structure, similar to the TRIS control. Conversely, hydrogels treated with trypsin showed no fluorescence, confirming the complete degradation of BSA by trypsin and underscoring the hydrogel's high specificity and sensitivity to trypsin enzymatic activity.

Supporting Movie 1.

Enzymatic Untying of "8" Shaped Hydrogels: A Time-Lapse Study of Temperature and Trypsin Concentration Effects. The video depicts a time-lapse sequence showcasing the transformative behavior of "8" shaped hydrogel samples composed of BSA-PEGDA (2-100 mM). These samples were immersed in various concentrations of trypsin solutions (0.01% and 0.25% w/v) and observed under different

temperature settings (4°C, 22°C, and 37°C). The footage reveals the intriguing process of the "8" shaped, knot-like hydrogel structures untying due to the enzymatic activity of trypsin. The rate at which the knots unravel is shown to depend on both the trypsin concentration and the ambient temperature, with higher levels of both resulting in a quicker transformation. The shape-morphing changes were meticulously documented using a GoPro 10 camera, configured to capture time-lapse footage at 10-second intervals, providing a clear and detailed visual record of the hydrogel behavior under enzymatic influence.

Supporting Movie 2.

Simultaneous Shape Transformation and Fluorescent Drug Release in a Hydrogel-Based Soft Actuator. An arc containing FITC was created using the same method as the active samples. The arc was immersed in 5 mL of trypsin (0.25% w/v) and placed in a UV camera (ChemiDoc Imaging System, Bio-Rad Company) at RT for one week, with photos taken at specific intervals. A consistent exposure time of 0.068 seconds was used. A simultaneous shape-morphing and decreased fluorescence intensity were observed, indicating that this hydrogel possesses dual functionalities of shape morphing and drug release.
